# Cargo selective vesicle tethering: The structural basis for binding of specific cargo proteins by the Golgi tether component TBC1D23

**DOI:** 10.1126/sciadv.adl0608

**Published:** 2024-03-29

**Authors:** Jérôme Cattin-Ortolá, Jonathan G. G. Kaufman, Alison K. Gillingham, Jane L. Wagstaff, Sew-Yeu Peak-Chew, Tim J. Stevens, Jérôme Boulanger, David J. Owen, Sean Munro

**Affiliations:** ^1^ MRC Laboratory of Molecular Biology, Francis Crick Avenue, Cambridge CB2 0QH, UK.; ^2^ Cambridge Institute for Medical Research, Cambridge Biomedical Campus, Hills Road, Cambridge CB2 0XY, UK.

## Abstract

The Golgi-localized golgins golgin-97 and golgin-245 capture transport vesicles arriving from endosomes via the protein TBC1D23. The amino-terminal domain of TBC1D23 binds to the golgins, and the carboxyl-terminal domain of TBC1D23 captures the vesicles, but how it recognizes specific vesicles was unclear. A search for binding partners of the carboxyl-terminal domain unexpectedly revealed direct binding to carboxypeptidase D and syntaxin-16, known cargo proteins of the captured vesicles. Binding is via a threonine-leucine-tyrosine (TLY) sequence present in both proteins next to an acidic cluster. A crystal structure reveals how this acidic TLY motif binds to TBC1D23. An acidic TLY motif is also present in the tails of other endosome-to-Golgi cargo, and these also bind TBC1D23. Structure-guided mutations in the carboxyl-terminal domain that disrupt motif binding in vitro also block vesicle capture in vivo. Thus, TBC1D23 attached to golgin-97 and golgin-245 captures vesicles by a previously undescribed mechanism: the recognition of a motif shared by cargo proteins carried by the vesicle.

## Teaser

A class of transport vesicle destined for the Golgi is recognized by a tether binding directly to the cargo it is carrying.

## INTRODUCTION

The transport of proteins between the organelles of the secretory and endocytic pathways is mediated by tubular/vesicular carriers that bud off donor organelles and then fuse with destination organelles. The process by which the vesicles recognize their correct destination involves tethering factors that mediate the initial capture of the vesicle before the SNARE proteins in the vesicle and destination membranes form a complex that then drives membrane fusion (*1*, *2*). Depending on the transport step, these tethering factors are long coiled-coil proteins or large protein complexes that are located to a specific organelle (*3*–*5*). In the case of the Golgi apparatus, these tethers include a set of long coiled-coil proteins called “golgins.” They are located to specific parts of the Golgi stack via C-terminal domains that either bind small guanosine triphosphatases (GTPases) or form a single transmembrane domain that spans the Golgi bilayer (*6*–*8*). Their role as tethers has been clearly demonstrated by the finding that replacing their C-terminal domains with one that directs targeting to mitochondria results in the accumulation of specific Golgi-destined carriers at this ectopic location (*9*–*11*).

**Fig. 1.**
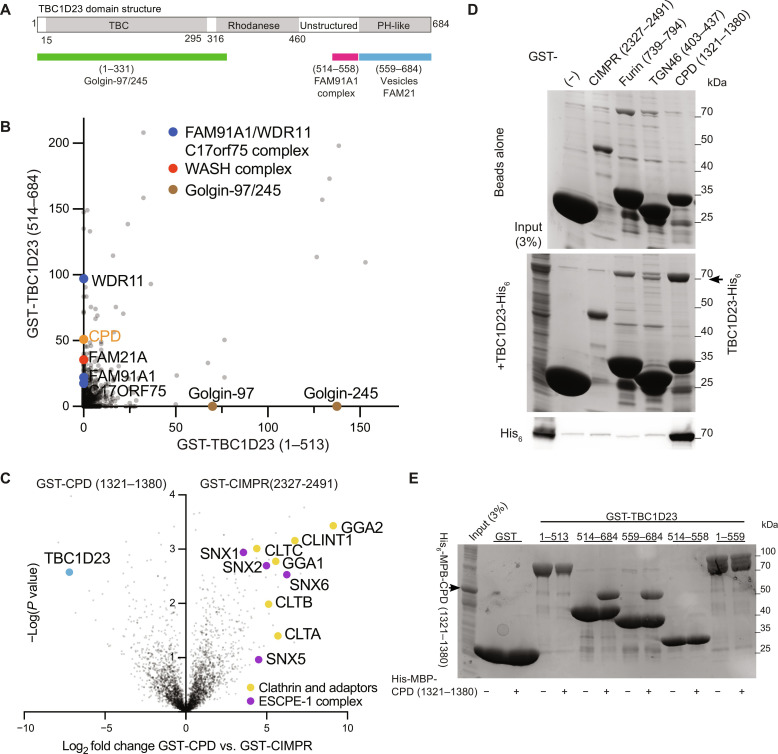
The C-terminal domain of TBC1D23 forms a complex with the cytoplasmic tail of CPD. (**A**) Domain structure of mouse TBC1D23 (UniProt Q8K0F1), the version of the protein used in this study. Labeling indicates the regions found by deletion mapping to bind the golgins, the FAM91A1 complex, the WASH complex, and vesicles (*12*, *14*). (**B**) MS analysis from affinity chromatography of 293T cell lysates using GST-TBC1D23 fragments. The plot compares the average spectral counts from two independent replicates of GST-TBC1D23 (1 to 513) versus GST-TBC1D23 (514 to 684). Values are in data S1. (**C**) Volcano plot of MS analysis comparing the eluates from affinity chromatography of 293T cell lysates using the cytoplasmic tails of CPD or CIMPR. The plot compares the spectral intensities from proteins bound to each bait, using data from three independent biological replicates. Endosomal sorting complex for promoting exit 1 (ESCPE-1). Values are in data S1. (**D**) Coomassie-stained gel and anti-His_6_ immunoblot showing that TBC1D23-His_6_ binds directly and specifically to the cytoplasmic tail of CPD. GST-tagged tails of indicated endocytic cargoes were immobilized on beads and incubated with bacterial lysate containing TBC1D23-His_6_. Representative of three repeats. (**E**) Coomassie-stained gel showing that the C-terminal domain of TBC1D23 is necessary and sufficient for binding to CPD. GST-tagged fragments of TBC1D23 were immobilized on beads and incubated with lysate from bacteria expressing the cytoplasmic tail of CPD [His_6_-MBP-CPD (1321 to 1380)]. Representative of two repeats.

The ability of golgins to capture vesicles raises the question of how these and other tethers can recognize the correct vesicles. In the case of the golgins, conserved N-terminal regions are necessary and sufficient to mediate vesicle capture (*10*). Three golgins, golgin-97, golgin-245, and GCC88, can capture endosome-to-Golgi carriers, and the first two share a closely related vesicle capture motif at the N terminus. This motif binds to a cytosolic protein, TBC1D23, a member of the Tre2/Bub2/Cdc16 (TBC) family of Rab GTPase-activating proteins (GAPs) (Fig. 1A). However, TBC1D23 is unlikely to have GAP activity as it lacks the conserved catalytic residues necessary for stimulating guanosine 5′-triphosphate hydrolysis (*12*, *13*). TBC1D23 is located to the Golgi in a manner that is dependent on the presence of golgin-97 and golgin-245, and it can, by itself, capture vesicles when relocated to mitochondria, indicating that it forms a bridge between the golgins and endosome-derived vesicles (*12*). In addition to the TBC domain, TBC1D23 has a rhodanese domain and a C-terminal domain that is related to pleckstrin homology (PH) domains (*13*, *14*). The TBC/rhodanese N-terminal region of the protein binds directly to the N terminus of golgin-97 or golgin-245, and the C-terminal domain is necessary and sufficient for vesicle capture (*12*, *14*). Between these two regions, there is a linker region, part of which binds to a complex of three proteins FAM91A1, WDR11, and C17orf75 (*12*, *15*, *16*). The function of this complex is unknown, and the part of TBC1D23 that it binds is not required for vesicle capture (*12*).

The ability of the C-terminal domain of TBC1D23 to capture a specific class of vesicles means that it must recognize a feature shared by these vesicles. Previous studies have shown that the C terminus can bind to the endosomal Wiskott-Aldrich syndrome protein and scar homolog (WASH) complex via the FAM21 subunit, which has a long C-terminal tail that also binds several other WASH interactors and regulators (*12*, *17*, *18*). However, WASH is thought to be involved in retromer- and retriever-based carrier formation at endosomes, including carriers that are destined for the Golgi and others destined for the plasma membrane (*19*, *20*). This led to the suggestion that TBC1D23 is able to bind to an additional factor that allows it to specifically capture carriers destined for the Golgi (*12*). Here, we describe the application of an unbiased affinity chromatography approach to identifying interaction partners of the C-terminal domain of TBC1D23 and report that it can bind directly to the cytoplasmic tails of several of cargo proteins that are found in endosome-to-Golgi carriers. This provides a mechanism by which TBC1D23 and its associated golgins can capture and tether endosome–derived carriers as they arrive at the Golgi.

## RESULTS

### TBC1D23 binds directly to the cytoplasmic tail of the endocytic cargo carboxypeptidase D

To identify binding partners for the C-terminal PH-like domain of TBC1D23, we immobilized on beads a glutathione *S*-transferase (GST) fusion to the domain and performed affinity chromatography of lysates from 293T cells. Mass spectrometry (MS) of the bound proteins revealed, as expected, that the C-terminal domain could selectively enrich the subunits of the FAM91A1 complex along with the WASH complex subunit FAM21, while the N-terminal domain bound to its partner golgins (Fig. 1B). As is typical for GST fusion–based purifications, the proteins bound to the C-terminal domain contained several abundant chaperones and cytosolic enzymes that are likely to reflect nonspecific interactions, but in addition to these, we identified carboxypeptidase D (CPD). This metalloprotease is broadly expressed and removes C-terminal basic residues following the action of furin and related proteases on diverse substrates including neuropeptides and growth factors (*21*, *22*). CPD is known to be primarily localized to the trans-Golgi network (TGN) and to recycle through endosomes and the plasma membrane. CPD is a type I protein with a 60-residue cytoplasmic tail, and so to validate this interaction, we used a GST fusion to the tail for affinity chromatography from 293T cell lysates. This showed that TBC1D23 has a strong preference for the tail of CPD over that of a control protein—the cation-independent mannose 6-phosphate receptor (CIMPR), another abundant protein that recycles between endosomes and the TGN (Fig. 1C). The interaction between TBC1D23 and CPD could be recapitulated with proteins expressed in *Escherichia coli*, demonstrating that it is direct (Fig. 1D), and use of truncated forms confirmed that it is the C-terminal domain of TBC1D23 that binds to the CPD tail (Fig. 1E). Together, these results show that the cytoplasmic tail of CPD can form a stable complex with residues 559 to 684 of TBC1D23, the C-terminal domain that mediates vesicle capture.

### TBC1D23 is required for the normal trafficking of CPD

**Fig. 2.**
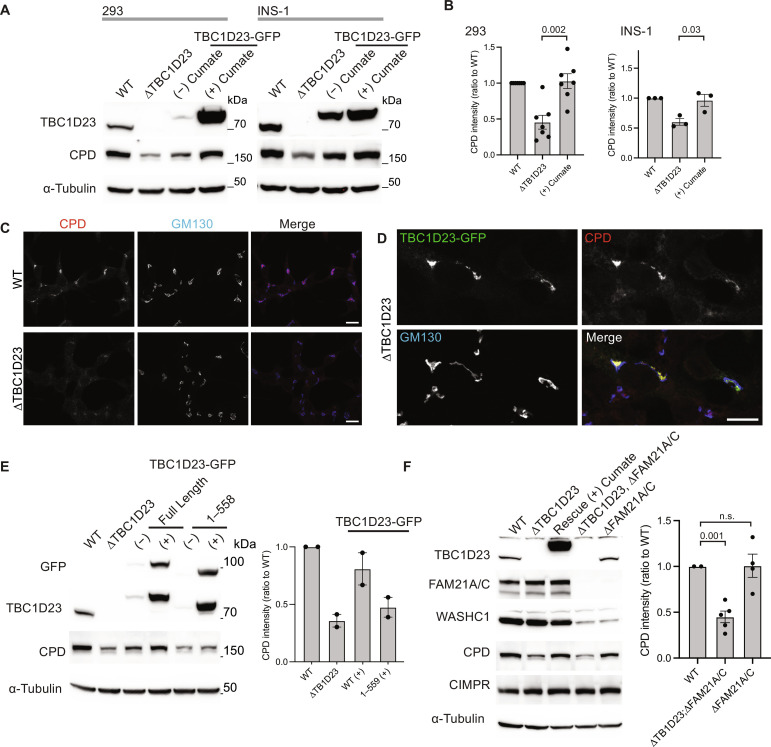
Normal CPD trafficking requires the C-terminal domain of TBC1D23 but does not require the WASH complex. (**A**) Immunoblots comparing TBC1D23, CPD, and α-tubulin in whole-cell lysates of HEK-293 and Ins-1 cells that were either wild type (WT), ∆*TBC1D23*, or the latter rescued with TBC1D23-GFP stably expressed under a cumate-inducible promoter as indicated. (**B**) The intensities of the bands in (A) were quantified and normalized to wild type (shown are mean and SEM, *P* values calculated using two-tailed unpaired *t* test); numbers of biological replicates are 6 (HEK-293) or 3 (INS-1). (**C**) Confocal micrographs of wild-type or ∆*TBC1D23* INS-1 cells stained for endogenous CPD and GM130 (Golgi marker). Scale bars, 10 μm. Representative of three repeats. (**D**) Confocal micrographs of *∆TBC1D23* INS-1 cells transiently expressing mouse TBC1D23-GFP and labeled for the GFP and endogenous CPD and GM130. Scale bar, 10 μm. Representative of three repeats. (**E**) Immunoblots of the indicated HEK-293 cell lines as in (A) but with the addition of HEK-293 cells expressing TBC1D23-GFP (1 to 558) and with quantification as in (B) showing mean and SEM of two independent replicates. Camera-based imaging of enhanced chemiluminescence from the secondary antibodies can result in very low background outside of the relevant bands. (**F**) Immunoblots of whole-cell lysates from wild-type HEK-293 cells (WT) or the indicated mutants, some of which expressed TBC1D23-GFP from a cumate-inducible promotor. CPD levels were quantified as in (B) for blots from three independent clones of *∆FAM21A/C* and *∆TBC1D23*;*∆FAM21A/C*, with mean values and SEM shown and *P* values calculated using two-tailed unpaired Welch’s *t* test. CPD is destabilized by removal of TBC1D23 but not by removal of FAM21A/C. Source data for (B), (E), and (F) are in data S2. n.s., not significant.

To test whether TBC1D23 is required for the trafficking of CPD, we used CRISPR-Cas9 to remove the *TBC1D23* gene from both human embryonic kidney (HEK) 293 cells and the rat insulinoma line INS-1 (Fig. 2A and fig. S1A). CPD cycles between endosomes and the Golgi, and for some such proteins, it has been found that perturbation of retrieval from endosomes results in their destabilization (*12*, *21*, *23*). In both knockout cell lines, the steady-state level of CPD was reduced, and this could be rescued by expression of TBC1D23–green fluorescent protein (GFP) from a stably transfected gene under the control of a cumate-inducible promotor (Fig. 2, A and B). In rat INS-1 cells, it was possible to detect CPD by immunofluorescence, and the levels of the protein in the TGN were reduced in the absence of TBC1D23 (Fig. 2C). Again, this phenotype was rescued by expression of TBC1D23-GFP but not a form lacking the C-terminal domain (Fig. 2, D and E, and fig. S1, B to D).

The C-terminal domain of TBC1D23 has been previously reported to bind to the FAM21 subunit of the WASH complex (*12*, *13*). However, removal of FAM21 from HEK-293 cells did not affect the steady-state levels of CPD (Fig. 2F). This indicates that the role of TBC1D23 in CPD traffic does not depend on the presence of FAM21. In contrast, deletion of the two golgins that bind TBC1D23 did reduce overall CPD levels as expected (fig. S1E). Together, these results show that TBC1D23 is required to maintain steady-state levels of CPD in the TGN and that performing this function involves an activity of TBC1D23 that is distinct from its ability to bind FAM21.

### Conserved residues in CPD mediate its interaction with TBC1D23

**Fig. 3.**
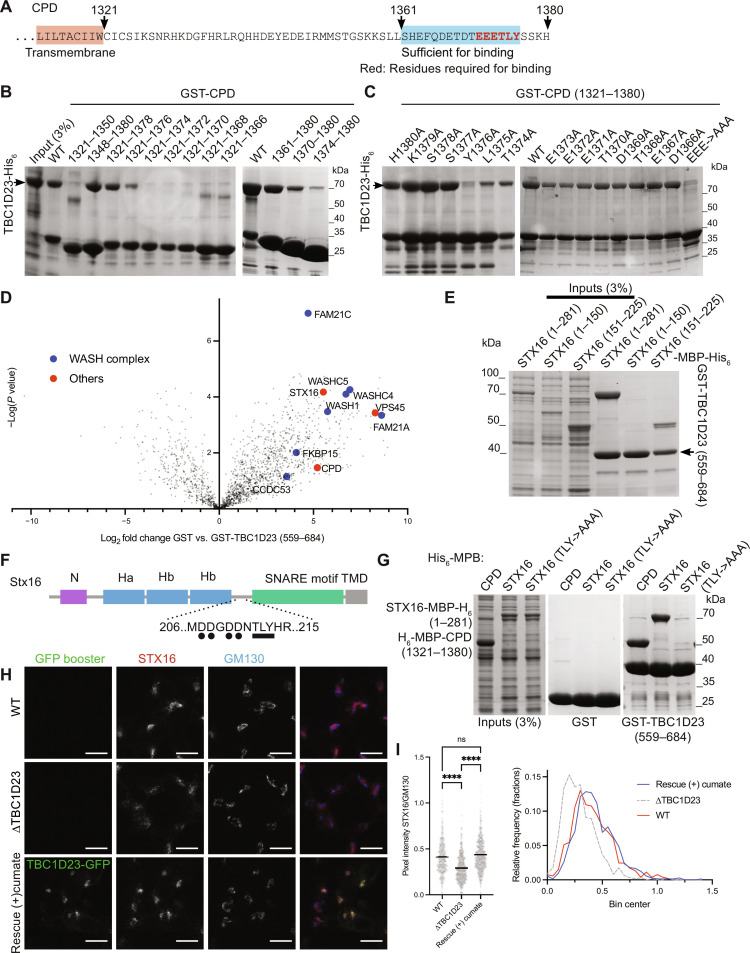
TBC1D23 binds to C-terminal conserved residues of the cytoplasmic tail of CPD. (**A**) The cytoplasmic tail of human CPD. (**B** and **C**) Coomassie-stained gels of the eluates from immobilized GST-CPD truncations or mutants incubated with a bacterial lysate containing TBC1D23-His_6_. Each representative of two repeats. (**D**) Volcano plot of the MS analysis from affinity chromatography of 293T cell lysates using bacterially expressed GST-TBC1D23 (559 to 684) or GST alone. Shown are mean spectral intensities of bound proteins from three independent experiments. Values are in data S1. (**E**) Coomassie-stained gel showing that the eluates from immobilized GST-TBC1D23 (559 to 684) incubated with lysates from bacteria expressing the indicated fragments of syntaxin-16 (STX16)–MBP–His_6_ (numbering as in UniProt O14662-2). Representative of two repeats. (**F**) Cartoon of syntaxin-16 showing the location of the key structural features and the acidic TLY motif related to that of CPD. (**G**) Coomassie-stained gel showing that the eluates from immobilized GST-TBC1D23 (559 to 684) incubated with lysates from bacteria expressing His_6_-MBP-CPD (1321 to 1380), syntaxin-16 (1 to 281)–MBP–His_6_ or syntaxin-16 (1-281 with T191A, L192A, and Y193A)–MBP–His_6_. Representative of two repeats. (**H**) Confocal micrographs of the indicated INS-1 cells (wild type, *∆TBC1D23*, and *∆TBC1D23* stably expressing TBC1D23-GFP under a cumate promoter in the presence of cumate for 24 to 36 hours). Cells stained for GFP and endogenous syntaxin-16 and GM130. (**I**) Scatter plot (left) showing the ratio of the Golgi fluorescence intensity of syntaxin-16 over the GM130-positive regions (Golgi). The horizontal bar is the mean. For wild type, *n* = 530, for *∆TBC1D23*, *n* = 628, and for the stable rescue, *n* = 725. ****P < 0.0001 [ordinary one-way analysis of variance (ANOVA) followed by a Sidak’s multiple comparison tests with a single pooled variance]. Ratios were also plotted as frequency distributions with a bin width of 0.05 (right). Values are in data S2.

To gain more understanding of the interaction between TBC1D23 and CPD, we mapped the region of the CPD tail that is required for binding and found that a C-terminal 16-residue region is necessary and sufficient (Fig. 3, A and B). The residues Leu^1375^ and Tyr^1376^ are particularly important, with upstream conserved acidic residues also contributing which becomes more apparent when several are mutated (Fig. 3, B and C). Nuclear magnetic resonance (NMR) spectroscopy of the CPD tail showed that the presence of TBC1D23 affected the residues in the C-terminal region around the acidic threonine-leucine-tyrosine (TLY) motif, confirming it binding to this region in solution (fig. S2).

### TBC1D23 binds to the cytoplasmic domain of multiple cargoes of endosome-derived vesicles

The finding that TBC1D23 can bind directly to the cytoplasmic tail of CPD, a protein that is known to recycle from endosomes back to the Golgi, raised the possibility that this interaction could mediate the capture of endosome-derived vesicles by TBC1D23. However, the capture of these vesicles by TBC1D23 ectopically relocated to mitochondria still occurred in cells from which the *CPD* gene had been deleted, indicating that binding to CPD is not sufficient to account for vesicle capture (fig. S3, A to C). To find further factors that might contribute to vesicle capture in addition to CPD, we used GST-TBC1D23 (559 to 684) for affinity chromatography of 293T cell lysates. As expected, we identified CPD and subunits of the WASH complex among the interacting proteins (Fig. 3D). Also enriched was the SNARE syntaxin-16, a known cargo of endosome-to-Golgi carriers, along with one of its interacting partners, the Sec1/Munc18-family protein VPS45 (*24*). We confirmed that syntaxin-16 is present in vesicles captured by either TBC1D23 or golgin-97 when they are relocated to mitochondria in HeLa cells (fig. S3, D and E). The syntaxin-16 cytoplasmic domain bound directly to the TBC1D23 C-terminal domain in vitro, and residues 151 to 225 of syntaxin-16 are both necessary and sufficient for the interaction (Fig. 3E). CPD binds to TBC1D23 via the sequence EEETLY, and syntaxin-16 contains a similar sequence: _209_DDNTLY in the exposed linker between the SNARE domain and the Habc domain (Fig. 3F). Mutating the TLY sequence in syntaxin-16 to AAA abolished its binding to TBC1D23 (Fig. 3G). While the steady-state levels of syntaxin-16 were not detectably altered in ∆*TBC1D23* cells (fig. S3F), the Golgi-localized pool of the protein was reproducibly reduced, and this could be rescued by expression of TBC1D23-GFP (Fig. 3, H and I, and fig. S3G). Together, our results show that TBC1D23 binds to the cytoplasmic tail of at least two proteins in the vesicles that it captures and thus raise the possibility that this capture could occur via direct and specific interaction with a subset of vesicle cargoes.

### X-ray crystallography of a complex between TBC1D23 and syntaxin-16 illuminates their interaction

**Fig. 4.**
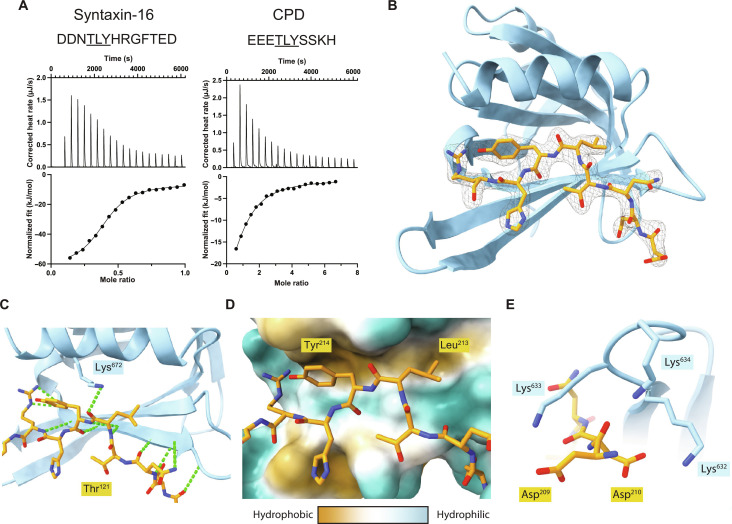
Structure of TBC1D23 C-terminal domain bound to a syntaxin-16 peptide. (**A**) ITC trace and fitted curve of the indicated peptides from syntaxin-16 (left) and CPD (right) binding to TBC1D23 C-terminal domain. Source data are in data S2. (**B**) Structure of the TBC123 C-terminal domain (sky blue) and syntaxin-16 (209 to 217) (gold) with the corresponding electron density map of the latter. (**C**) Close-up of the hydrogen bonding network between syntaxin-16 (gold) and TBC1D23 (sky blue). Lys^672^ hydrogen bonds to the backbone of syntaxin-16. Hydrogen bonds are in green. (**D**) Hydrophobicity surface plot of TBC1D23 showing the packing of residues of syntaxin-16 (gold) into two hydrophobic pockets. (**E**) Close-up of Asp^209^ and Asp^210^ in syntaxin-16 (gold) interacting with Lys^632^ to Lys^634^ in TBC1D23 (sky blue).

To understand how the C-terminal domain of TBC1D23 recognizes the tails of vesicle cargo, we used a combination of x-ray crystallography and biochemistry. Peptides corresponding to the relevant regions of syntaxin-16 and CPD showed robust enthalpic-dominated binding to the C-terminal domain by isothermal titration calorimetry (ITC) with affinities of ~1.4 and 10.6 μM, respectively, and a stoichiometry of 1:1 (Fig. 4A). Crystallization of the C-terminal domain in the presence of the syntaxin-16 peptide yielded crystals that diffracted to 3.3-Å resolution and were solved by molecular replacement using the previously published apo structure (6JM5) in space group *P*6_2_22 (table S1). Four C-terminal domains were present within the asymmetric unit (fig. S4, A to C). Two of the domains dimerized via strand exchange in the same manner as that seen in the peptide-free structure with reciprocal C-terminal tails (VLDALES) inserting between strand 5 and the helix 2 of the other domain but with no sign of the peptide (*13*). In the third domain, the electron density was unclear, but for the final domain, density corresponding to a single syntaxin-16 peptide was visible (fig. S4D). This peptide density was in the same position that was occupied by the cross-dimerizing, C-terminal tail in the first two molecules of the asymmetric unit.

Given the above results, we generated a version of the C-terminal domain lacking the last seven residues (VLDALES). Crystals of this truncated domain in the presence of the syntaxin-16 peptide diffracted to higher resolution (2.2 Å) and were solved in space group *P*6_5_22 again by molecular replacement with unliganded 6JM5 (table S1). The asymmetric unit contained two copies of the domain with both having density corresponding unambiguously to the syntaxin-16 peptide in the previously proposed binding site (fig. S4E). The previously observed strand-exchanged dimer was absent with the homodimeric C-terminal tail–mediated interface being replaced by the peptide. Thus, the structure elucidates how the syntaxin-16 peptide binds to the C-terminal domain of TBC1D23 (Fig. 4B). The peptide extends TBC1D23’s β sheet by adopting a twisted β augmentation mode of interaction with strand 5 and buries ~670 Å^2^ of the C-terminal domain’s solvent accessible surface area, a similar sized interface to those seen previously for other trafficking motif–binding proteins that have a similar *K*_D_ of interaction (*25*, *26*). The Thr^212^ in the acidic TLY motif of syntaxin-16 packs its methyl group against the alkyl chain of Lys^628^ that orients the hydroxyl group toward the solvent to stabilize a somewhat strained backbone conformation (Fig. 4C). This allows the side chains of the leucine and tyrosine residues to be splayed apart and simultaneously buried into neighboring hydrophobic pockets in the domain (Fig. 4D). As a result, the acidic residues Asp^209^ and Asp^210^ of syntaxin-16 are in close proximity to three basic residues (Lys^632^ to Lys^634^) in the C-terminal domain of TBC1D23, which would allow a direct electrostatic interaction (Fig. 4E).

### Mutations in the TBC1D23 C-terminal domain and the syntaxin-16 peptide perturb their interaction

**Fig. 5.**
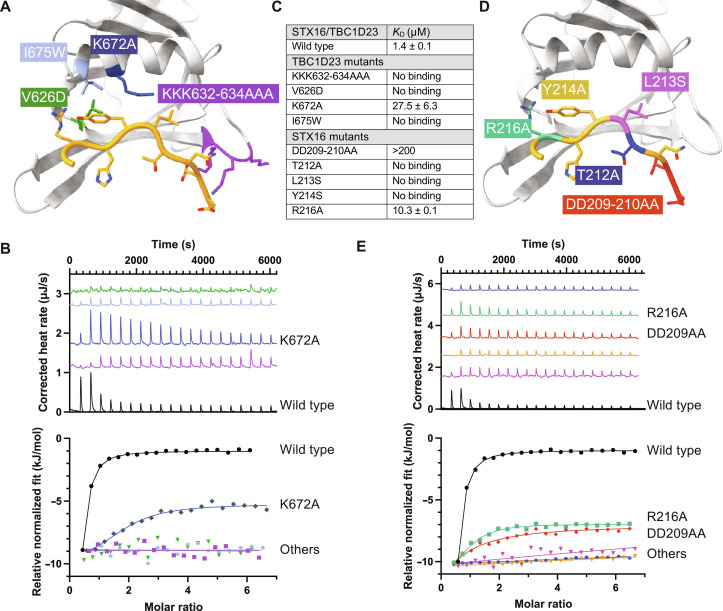
Mutational analysis of the interaction between TBC1D23 and syntaxin-16. (**A**) Schematic view of the mutations in TBC1D23 that were tested by ITC. (**B**) ITC of relative binding of syntaxin-16 peptide to the TBC1D23 C-terminal domain mutants. Mutations are indicated by the color coding in (A), with wild type in black. (**C**) Summary of ITC results. Source data are in data S2. (**D**) Schematic view of the mutations in the syntaxin-16 peptide that were tested by ITC. (**E**) ITC of relative binding of the mutant syntaxin-16 peptides to the TBC1D23 C-terminal domain. Mutations are indicated by the color coding in (D), with wild type in black.

The structure indicates that recognition of the acidic cluster and the TLY triad are the critical factors in driving peptide binding and specificity. To validate this, a series of point mutations was designed in the C-terminal domain and initially assessed for folding by yield and circular dichroism. Two of the residues that form parts of the hydrophobic pockets that bind the leucine and tyrosine of the peptide (Ile^629^ and Ile^639^) are also part of the domain’s core hydrophobic residues, and their mutation caused misfolding (I629S and I639S). The remaining mutants were assayed for binding to the syntaxin-16 peptide by ITC (Fig. 5, A to C). Lys^672^ of TBC1D23 hydrogen bonds via its amine group to the carboxyl peptide bond of Lys^213^ in the TLY in syntaxin-16 (Fig. 4C); mutation to alanine (K672A) weakened binding ~20 fold (*K*_D_ ~ 30 μm). Mutation of Val^626^, which packs against the aromatic ring of reside Tyr^214^, to aspartate (V626D) abolishes peptide binding. The binding pocket in TBC1D23 for Tyr^214^ was obstructed by mutating one of its lining residues (I675W), and, again, this reduced binding to below detectable levels (*K*_D_ > 300 μM). Last, mutation to alanine of Lys^632^, Lys^633^ and Lys^634^, which are adjacent to the TLY-binding hydrophobic grove, also abolished peptide binding, indicating that their interaction with the acidic cluster in syntaxin-16 is important. Thus, mutations that either remove interactions with the peptide or protrude into the binding pocket strongly affect peptide binding, confirming that the interactions seen in the crystal also occur in solution.

Mutations were also made in the syntaxin-16 peptide to further validate the interactions observed in the crystal structure (Fig. 5D). ITC assays confirmed a role in binding for all three of the residues in the acidic TLY motif and for the two adjacent acidic residues, with Arg^216^ making a smaller contribution (Fig. 5, C to E). The acidic TLY motif is absolutely conserved between syntaxin-16 and CPD, and so to determine whether conservative changes in the motif can be tolerated, we tested variants of these three residues (fig. S4F). Mutation of Thr^212^, which holds the leucine and tyrosine in a strained position to allow engagement with their pockets, to serine (T212S) reduced binding ~20-fold to *K*_D_ ~ 30 μM. Changing Lys^213^ to the smaller alanine (L213A) or the larger phenylalanine (L213F) both caused a reduction of binding of >100-fold, consistent with the side-chain fitting into a size-specific hydrophobic pocket in the structure, i.e., a medium-sized hydrophobic residue is required at this position. Last, Tyr^214^ is more tolerant of conservative mutation with Y214W and Y214F peptides having *K*_D_ values of ~7 and ~15 μM, respectively (fig. S4F).

### Further binding partners for TBC1D23 are identified by use of the C-terminal domain–binding motif

**Fig. 6.**
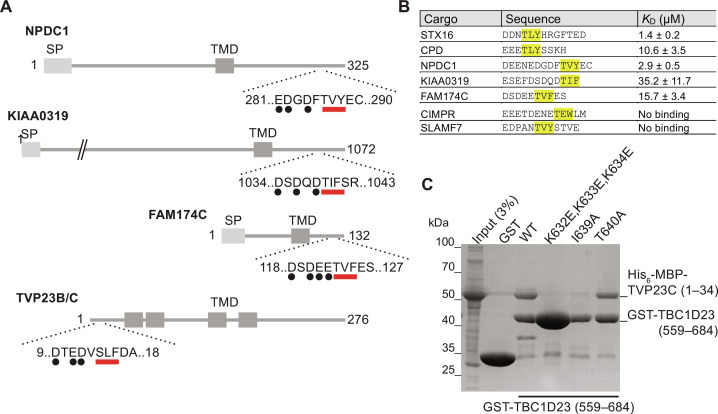
Identification of membrane proteins containing a TBC1D23-binding acidic TLY motif. (**A**) Schematic of four human proteins that have an acidic TLY motif in their cytoplasmic tail and that have been reported to recycle from endosomes to the Golgi or be localized to the Golgi (SP, signal peptide; TMD transmembrane domain). (**B**) Binding affinities of the indicated peptides containing the acidic TLY motifs from the proteins shown in (A), as determined by ITC (fig. S5; source data are in data S2.). (**C**) Coomassie-stained gel showing the binding of the N-terminal cytoplasmic tail of TVP23C [His_6_-MBP-TVP23C (1 to 34)] to beads coated with the C-terminal domain of TBC1D23 fused to GST. Mutations in the acidic TLY motif disrupt the interaction. Representative of two repeats.

Although both syntaxin-16 and CPD bind to the C-terminal domain through a closely related motif, the only functional property that they share is the fact that they are both in vesicles that recycle between endosomes and Golgi and can be captured by TBC1D23. We thus wondered whether there were further proteins in these vesicles that also have an acidic TLY motif that can bind TBC1D23. Peptide motifs that are recognized by proteins are rarely invariant, and so other versions of the motif are likely to be functional. From the above mutagenesis experiments and the nature of the interactions seen in the structure, we chose [DE]_> = 3/5_[TS][LIV][YFW] as being a loose but plausible definition of the core binding motif for the TBC1D23 C-terminal domain and used this to search the predicted cytoplasmic tails of all membrane proteins in the human proteome. In addition, we looked for membrane proteins among the interactors reported for TBC1D23 in the BioPlex protein interaction screen (*27*). The latter approach identified four membrane proteins, three of which are reported to be in the endosome/Golgi system and one of unknown location. The former approach identified 19 membrane proteins of which 10 are reported to be endosomal or Golgi, with two proteins found by both approaches (table S2). We thus tested the ability of the TBC1D23 C-terminal domain to bind to peptides corresponding to the TLY-like region from several of these proteins (Fig. 6, A to C, and fig. S5).

NPDC1 is a largely uncharacterized protein suggested to play a role in neuropeptide secretion and dense core vesicle traffic (*28*, *29*), and the peptide from its tail has an affinity for TBC1D23 similar to that of syntaxin-16 at ~3 μM (fig. S5). KIAA0319 is a neuronal protein linked to dyslexia, and although its function remains unknown, it has been reported to recycle from the surface back to the Golgi region (*30*). The peptide from KIAA0319 showed weak but detectable binding with *K*_D_ ~ 35 μM (fig. S5). KIAA0319 is only expressed in neuronal cells but KIAA0319L, its non-neuronal paralog, is enriched in vesicles captured by golgin-97 and its localization is perturbed in human cells lines and zebrafish lacking TBC1D23 (*31*, *32*). The presence of the TBC1D23-binding motif in these neuronal proteins may be relevant to human mutations in TBC1D23 causing defects in neurodevelopment (*33*, *34*). FAM174A and its paralogs FAM174B and FAM174C are small-membrane proteins of unknown function that have been reported to be in the Golgi (*35*). All have a TLY-like motif and the peptide from FAM174C bound with an affinity of 16 μM. Last, TVP23B/C is a small polytopic membrane protein known to recycle between endosomes and Golgi and to be enriched in vesicles captured by golgin-97 (*32*, *36*), and its 34-residue cytoplasmic tail bound to the TBC1D23 C-terminal domain with this interaction disrupted by mutating the three lysine residues in TBC1D23 that we have shown to be required for binding CPD and syntaxin-16 (Fig. 6C). In contrast, no binding was seen with peptides from CIMPR, which also traffics between Golgi and endosomes and has an acidic region in its tail but no TLY-like motif, and SLAMF7 that has a TLY-like motif but a smaller acidic cluster that contains a proline (Fig. 6B). Thus, multiple proteins that recycle between endosomes and Golgi have sequences in their cytoplasmic tails that can bind directly to the C-terminal domain of TBC1D23.

### Residues in TBC1D23 that are required for binding the acidic TLY motif are also required for vesicle capture in vivo

**Fig. 7.**
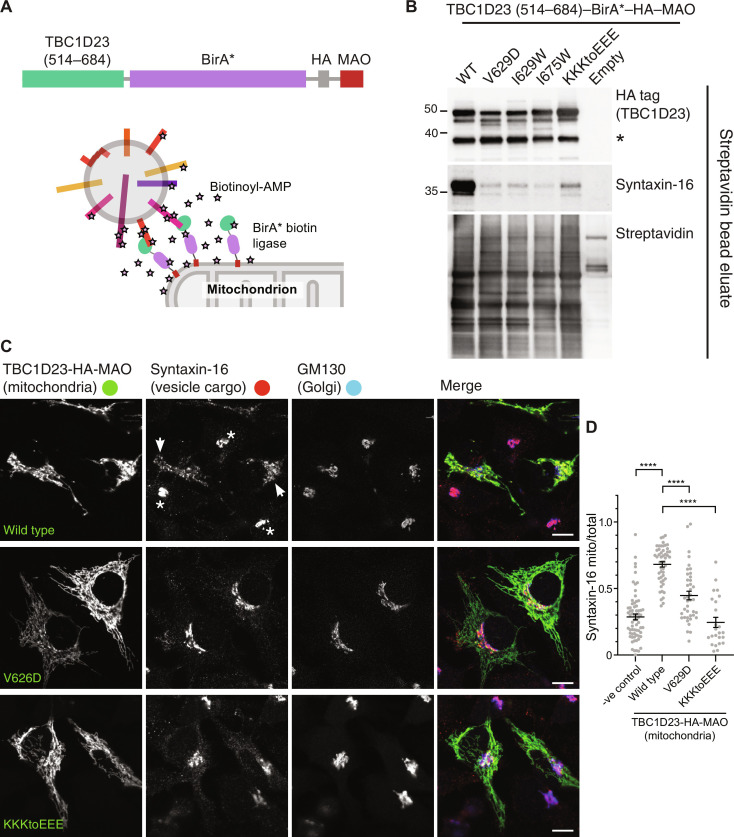
Residues in TBC1D23 required for peptide binding in vitro are also required for vesicle capture in vivo. (**A**) Ectopic relocation and biotinylation: a chimera comprising the TBC1D23 C-terminal domain attached to the BirA* promiscuous biotin ligase and the mitochondrial targeting signal from monoamine oxidase (MAO). (**B**) Immunoblots of streptavidin precipitations from whole-cell lysates of 293T cells transfected with the indicated variants of the mitochondrially targeted C-terminal domain of TBC1D23 fused to BirA*. Mutation of key residues does not affect total biotinylation or the stability of the chimera but greatly reduces biotinylation of syntaxin-16 indicating loss of vesicle capture. The lower band (asterisk) indicates some clipping of the chimera apparently between the TBC1D23 part and BirA*. Representative of three repeats. (**C**) Immunofluorescence of cells expressing the indicated forms of mitochondrial full-length TBC1D23 and immunolabeled for the TBC1D23 chimera [hemagglutinin (HA) tag], syntaxin-16, and the Golgi marker GM130. TBC1D23 is sufficient to cause the accumulation of vesicles at this ectopic location, and, hence, syntaxin-16 accumulates on mitochondria coated in wild-type TBC1D23 (arrows), rather than being predominantly in the Golgi in untransfected cells (asterisks). The mutations in TBC1D23 that disrupt peptide binding in vitro greatly reduce mitochondrial accumulation of syntaxin-16. Representative of three repeats. Scale bars, 10 μm. (**D**) Quantitation of the degree of mitochondrial relocation of syntaxin-16 in (C). The relocation induced by the wild-type protein, and the reductions in this relocation caused by the mutations are statistically significant (****P < 0.0001, unpaired, two-tailed *t* tests).

The presence in endosome-to-Golgi vesicles of diverse proteins with a TLY-like motif in their cytoplasmic tails raises the possibility that TBC1D23 can capture these vesicles by binding to the tails protruding from the vesicle. TBC1D23 is sufficient to capture vesicles when relocated to mitochondria (*12*), and so we tested the effect on this capture of mutations that disrupt peptide binding in vitro. Relocation of vesicles by the constructs was quantified with proximity biotinylation (BioID) using a promiscuous biotin ligase fused to TBC1D23 along with a mitochondrial targeting signal (Fig. 7A). When wild-type TBC1D23 was expressed on mitochondria, vesicle cargo was efficiently biotinylated as expected (Fig. 7B). However, mutation of the residues that disrupt peptide binding in vitro resulted in a near complete loss of biotinylation of vesicle cargo proteins, indicating that vesicle capture was also disrupted. This is not simply due to the vesicle being docked by a different mechanism, as immunofluorescence demonstrated that the accumulation of vesicle cargo proteins on the TBC1D23-coated mitochondria was greatly reduced (Fig. 7, C and D). Together, these results demonstrate that the same part of TBC1D23 that binds to the tails of vesicle cargo in vitro is required for vesicle capture in vivo, consistent with a model in which TBC1D23 can capture the incoming vesicle by recognizing its cargo proteins.

## DISCUSSION

The C-terminal domain of TBC1D23 was known to be sufficient to capture one or more of the classes of carrier that mediate transport of membrane proteins from endosomes to Golgi (*12*, *13*). Our search for binding partners of this domain has now revealed that it can bind directly to the cytoplasmic tails of several of the cargo proteins that are present in these vesicles via a conserved acidic TLY motif. This interaction provides a mechanism by which TBC1D23 displayed on the ends of golgin-97 and golgin-245 can recognize specific vesicles and thus tether them to the trans-Golgi before subsequent SNARE engagement and vesicle fusion. For both CPD and syntaxin-16, their predicted structures indicate that the acidic TLY motif is likely to be located at some distance from the transmembrane domain and, hence, the vesicle membrane. The unstructured tail of CPD or the α-helical bundle of syntaxin-16 are predicted to place the motif ~15 nm away from the membrane which would allow recognition to be unimpeded by the vesicle surface.

The direct binding of a tethering factor to the cargo carried by a vesicle has not been previously proposed as a mechanism for vesicle capture, but it would certainly ensure that only a specific set of vesicles is recognized (*37*). Like all such tethering interactions, the mechanism requires that the tether binds only to the vesicle and not to the organelle from which the cargo-laden vesicle budded. It is possible to imagine several ways in which this could be achieved. TBC1D23 is primarily localized at the Golgi as determined by both immunofluorescence and organelle fractionation, indicating that the interaction of its N-terminal domain with golgin-97 and golgin-245 is the dominant factor in determining its localization in vivo (*12*, *32*, *33*). In addition, the golgins are homodimers, and so each golgin is likely to display two copies of TBC1D23 that would increase the avidity of the interaction with the tails in the vesicle over that of a monomer of TBC1D23 that was free in the cytoplasm. It may be that interaction between TBC1D23 and the cargo proteins is only strong enough to capture a vesicle when an array of TBC1D23 presented on multiple golgins can interact with multiple TLY-containing proteins that are concentrated in a single vesicle, and, hence, the avidity of the interaction is increased even further.

Another mechanism that could account for TBC1D23 only recognizing cargo proteins that are present in vesicles is that the acidic TLY motif in the cargo proteins could be masked in some way when the cargo is in endosomes and other compartments. This could occur if the coat proteins that sort them into the retrograde pathway rapidly sequester them into forming carriers and if the interaction with the coat prevents recognition of the acidic TLY motif by TBC1D23 until the carrier has budded and uncoated. The identity of the coat that makes the carriers captured by TBC1D23 is as yet unresolved as there are multiple routes back from endosomes to the Golgi, with both clathrin coats with the activating protein 1 (AP-1) adaptor and sorting nexins with retromer proposed to be involved in generating carriers (*19*, *38*). Nonetheless, note that the acidic TLY motif includes a stretch of acidic residues, and acidic clusters can act as sorting signals for packaging into clathrin/AP-1–coated carriers (*39*, *40*). Both CPD and syntaxin-16 are known to be enriched in AP-1–dependent clathrin–coated vesicles, although whether these vesicles are delivering cargo to or from the trans-Golgi remains to be resolved (*41*).

There are known to be multiple classes of vesicle arriving at the Golgi from endosomes, and although TBC1D23 appears to responsible for the capture of at least some of these, the mechanism by which the others are captured is unknown. The golgin GCC88 captures at least one such set of vesicles although it does not bind TBC1D23, but how this is achieved is still unclear (*9*, *12*). This multiplicity of endosome-to-Golgi routes with only some carriers being captured by TBC1D23 is consistent with human mutation of TBC1D23 resulting primarily in neurodevelopmental defects despite being widely expressed in most tissues (*33*, *34*). It may be that when TBC1D23-dependent tethering is lost, other capture mechanisms can partially compensate, or some cargo proteins can return to the Golgi by some of the alternative routes.

The enrichment of a particular set of proteins in each specific type of transport vesicle is only one type of distinguishing feature that would allow the specific recognition necessary to ensure the fidelity of intracellular traffic. For most trafficking steps, vesicle recognition remains poorly understood. In some cases, Arf or Rab GTPases are believed to play a role, although they are also likely to be present on donor organelles resulting in the same issues of vesicle versus donor organelle identity (*4*, *37*, *42*). Our finding that a tethering factor can recognize a set of cargo proteins in the carriers that it captures not only sheds light on endosome-to-Golgi traffic but could also illuminate other membrane trafficking routes where the mechanism of vesicle recognition remains to be found.

## MATERIALS AND METHODS

### Plasmids

Details of the plasmids used in this report, together with the cloning methods used to generate them, are provided in data S3. Note that the cDNA for *TBC1D23* used throughout is from mouse (Q8K0F1) with 684 residues [corresponding to the 684-residue human isoform (UniProt Q9NU8-2)], and residues are numbered accordingly. Peptides for binding partners are all based on the human proteins.

### Antibodies

Full lists of primary and secondary antibodies used for Western blotting and immunofluorescence are provided in data S3.

### Mammalian cell culture

The full list of cell lines used are in data S3. HeLa (American Type Culture Collection), HEK293T (American Type Culture Collection, CRL-3216; referred to throughout the paper as 293T), and HEK-293 Flp-In T-REx 293 cells stably expressing Cas9 (referred to throughout the paper as HEK-293) were cultured in Dulbecco’s modified Eagle’s medium GlutaMAX (Gibco) supplemented with 10% fetal bovine serum (FBS) and penicillin/streptomycin at 37°C and 5% CO_2_. The insulinoma INS-1–derived 832/13 rat cell line was obtained from C. Newgard (Duke University School of Medicine) via M. Ailion (University of Washington, Seattle) (referred to throughout the paper as INS-1) (*43*). INS-1 cells were grown with RPMI-1640 GlutaMAX (Gibco), supplemented with 10% FBS, 1 mM sodium pyruvate, 10 mM Hepes, and penicillin/streptomycin at 37°C and 5% CO_2_. For transient transections, unless noted, we used polyethylenimine (PEI; Polyscience, 24765) dissolved in phosphate-buffered saline (PBS) to 1 mg/ml. The ratio of PEI (in microliters) to DNA (in micrograms) used was 3:1. PEI was dissolved in Opti-MEM (Gibco) and incubated at room temperature for 5 min. DNA was added and incubated for another 20 min at room temperature before dropwise addition onto cells that had been seeded the day before. Cells were free of mycoplasma as determined by routine testing using MycoAlert (Lonza).

### Generation of knockout cells using CRISPR-Cas9

Precise information about the cell lines generated and guide RNA (gRNA) sequences is provided in data S3. ∆*TBC1D23* (in HEK293 Flp-In T-REx 293 cell line stably expressing Cas9, HEK-293) cells were generated as follows: cells grown to ~70% confluence in six-well plates and cotransfected with a gRNA to *TBC1D23* (*12*) and pmaxGFP using Fugene6 (Promega) according to the manufacturer’s instructions. Forty-eight hours later, cells were trypsinized, and single GFP-positive cells were sorted into each well of 96-well plates using a cell sorter. Single colonies that grew were expanded and analyzed by immunoblotting.

All other knockouts made in HEK-293 and INS-1 cells were generated as follows: gRNA with high-efficiency and high-specificity scores was chosen using the UCSC genome browser and CRISPOR (*44*). To improve the knockout efficiency, each gene was targeted with two gRNA, one targeting an early exon and the second targeting a late exon, so a large part of the coding region could be removed (data S3). All gRNAs were cloned onto the pX459 vector, which also expresses Cas9 and a puromycin resistance gene (*45*). Cells were grown to ~70% confluence in six-well plates and transfected with a mixture of 6 μl of PEI and an equal amount of each vector coding for gRNAs for a total of 2 μg of DNA in 100 μl of Opti-MEM. Forty-eight hours later, the cells were trypsinized and replated in complete medium containing puromycin (1.5 μg/ml). Forty-eight to 72 hours later, cells were diluted to one cell per two wells in three 96-well plates and grown in complete medium. Clones that grew were expanded and screened by immunoblotting or immunofluorescence. When possible, several clones were assayed (data S3).

For rescue of knockout cell lines, the relevant genes were cloned into a piggyBac vector containing a puromycin resistance gene and the protein of interest expressed under a cumate-inducible promoter. Cells grown to ~70% confluence in 10-cm plates were cotransfected with the plasmid coding for the rescue constructs and the plasmid coding the piggyBac transposase at 5:1 molar ratio using 6 μg of DNA and 18 μl of PEI in 1 ml of Opti-MEM. Forty-eight hours after transfection, cells were trypsinized and replated in selection medium containing puromycin (1.5 μg/ml). The medium was changed every 48 to 72 hours while keeping the puromycin selection until confluence was reached (usually 10 days), and expression of the rescue construct was verified by Western blotting and immunofluorescence. Rescue lines were maintained as a polyclonal population.

### Immunofluorescence

Cells were transfected in 24-well plates (300 ng of DNA and 1 μl of PEI in 50 μl Opti-MEM) or in 6-well plates (1 μg of DNA and 3 μl of PEI in 100 μl of Opti-MEM). The next day, cells were dissociated using trypsin and seeded onto polytetrafluoroethylene-coated multiwell slides (Hendley-Essex). About 36 to 48 hours after transfection, cells were fixed with 4% paraformaldehyde in PBS (10 min), permeabilized in 0.5% (v/v) of Triton X-100 in PBS (5 min), and blocked for 1 hour in PBS containing 20% FBS and 0.25% Tween 20. Primary and secondary antibodies were applied sequentially in blocking buffer for 1 hour at room temperature. After washing, cells were mounted using ProLong Gold antifade mountant (Thermo Fisher Scientific). Slides were imaged using a Leica TCS SP8 laser scanning confocal microscope and a 63× lens.

### Quantification of fluorescent micrographs

For each condition in each independent replicate, three multichannel fluorescence micrographs at 1024 × 1024 resolution were acquired. The images were then automatically processed using a custom ImageJ macro to measure the intensity in all channels for regions of interest (ROIs) defined by segmenting the second channel (Golgi marker GM130). Briefly, the second channel was processed with a rolling ball and a median filter. A threshold based on the mean and SD of the image allowed the separation of the background and foreground, while a filter on minimum area removed spurious detection. ROIs were enlarged to include the nearby region. Last, the macro reports the area of the ROI and the mean and maximum value of each channel. For each condition, over 100 individual cells were quantified. For each ROI, the mean fluorescence intensity of the protein of interest was divided by the mean fluorescence intensity of the GM130 channel. The data of the ratio of mean fluorescence intensities were then plotted as frequency distributions using GraphPad Prism 9 with a bin width of 0.05. For each marker, the experiment was repeated twice with similar results.

### Determination of protein levels by immunoblotting

HEK-293 or INS-1 cells were seeded in 10-cm plates, and where appropriate, the medium was supplemented with 1× cumate (System Biosciences, QM159A-1) to induce expression of the rescue constructs. After 24 to 36 hours, cells were resuspended by scraping, washed twice with ice-cold PBS, and pelleted by a 5-min 500*g* spin at 4°C. Cells were resuspended in lysis buffer [50 mM tris (pH 7.4), 150 mM NaCl, 1 mM EDTA, 1% Triton X-100, and protease inhibitor cocktail (cOmplete, Roche)], and incubated on ice for 5 min. Lysates were clarified at 17,000*g* for 5 min at 4°C, and the total protein concentration was determined using the bicinchoninic acid assay (Thermo Fisher Scientific, 23227). Clarified lysates were then supplemented with 4× NuPAGE LDS sample buffer containing 100 mM dithiothreitol (DTT; Invitrogen, NP0007). Equal amount of total protein was separated by SDS–polyacrylamide gel electrophoresis (PAGE) using precast gels (Invitrogen, XP04205) and analyzed by immunoblotting.

### Immunoblotting

Protein samples in 1× NuPAGE LDS sample buffer containing 25 mM DTT were boiled at 95°C, loaded on to SDS-PAGE gels, and transferred to nitrocellulose membranes. Membranes were blocked in 5% (w/v) of milk in PBS-T [PBS with 0.1% (v/v) of Tween 20] for 1 hour, incubated overnight at 4°C with primary antibody in the same blocking solution, washed three times with PBS-T for 5 min, incubated with horseradish peroxidase (HRP)–conjugated secondary antibody in 5% (w/v) of milk in PBS-T for 1 hour and, washed three times with PBS-T for 5 min. Blots stained with HRP-conjugated secondary antibodies were imaged using a Bio-Rad ChemiDoc imager with SuperSignal West Pico PLUS (Thermo Fisher Scientific, 34577). The “gels” analysis tool in ImageJ was used for the quantification of blots, with raw data normalized to the wild-type band (data S2).

### In vitro binding assays using recombinant proteins

Recombinant proteins for binding assays were expressed as follows: Plasmids were transformed into *E. coli* BL21–CodonPlus (DE3)–RIL (Agilent, 230245). From an overnight starter culture, cells were grown in 2× TY medium containing ampicillin [100 μg/ml; or kanamycin (50 μg/ml) when appropriate] and chloramphenicol (34 μg/ml) at 37°C in a shaking incubator. When the culture reached optical density at 600 nm (OD_600_) = 0.6 to 0.8, the temperature was lowered to 16°C, and protein expression was induced with 100 μM isopropyl-β-d-1-thiogalactopyranoside (IPTG), and incubated overnight. Bacteria cells were harvested by centrifugation at 4000*g* at 4°C for 15 min and were mechanically resuspended on ice in lysis buffer containing 50 mM tris (pH 7.4), 150 mM NaCl, 1 mM EDTA, 5 mM 2-mercaptoethanol, 1% Triton X-100, and protease inhibitor cocktail (cOmplete, Roche). Cells were lysed by sonication, and the lysates were clarified by centrifugation at 20,000*g* at 4°C for 15 min. Clarified lysates were flash-frozen in liquid nitrogen and stored at −80°C until needed.

For binding to beads, saturating amounts of clarified bacterial lysates containing GST-tagged baits were added to glutathione-Sepharose beads previously washed with lysis buffer [50 mM tris (pH 7.4), 150 mM NaCl, 1 mM EDTA, 5 mM 2-mercaptoethanol, and 1% Triton X-100] and incubated at 4°C for 1 hour on a tube roller. Beads were washed once with lysis buffer, once with lysis buffer supplemented with 500 mM NaCl, and once again with lysis buffer and incubated with clarified bacterial lysates containing the recombinant prey for 2 hours at 4°C on a rotator. Beads were washed three times with lysis buffer and eluted by boiling in lysis buffer supplemented with a 4× solution of NuPAGE LDS sample buffer containing 100 mM DTT. Boiled slurry was separated on SDS-PAGE gels and analyzed by Coomassie blue stain (Abcam, ab119211) or by immunoblot using an anti-His_6_ HRP-conjugated antibody.

### TBC1D23 affinity chromatography from 293T cell lysate

GST-TBC1D23 (1 to 513) and GST-TBC1D23 (514 to 684) were expressed in the *E. coli* strain BL21-GOLD (DE3; Agilent Technologies). Bacteria were grown at 37°C to an OD_600_ of 0.7 and induced with 100 μM IPTG overnight at 16°C. Cells were harvested by centrifugation, resuspended in lysis buffer [25 mM tris-HCl (pH 7.4), 150 mM NaCl, 1 mM EDTA, 1% (v/v) Triton X-100, and one EDTA-free cOmplete protease tablet/50 ml and 1 mM phenylmethylsulfonyl fluoride (PMSF)], dounce-homogenized and sonicated on ice. The lysates were clarified by centrifugation at 12,000*g* for 15 min at 4°C and applied at saturating levels to glutathione-Sepharose beads. The beads were then incubated with cell lysates prepared from two confluent 175-cm^2^ flasks of 293T cells. 293T cells were collected by centrifugation at 500*g* for 3 min, washed once in ice-cold PBS, and lysed in lysis buffer [25 mM tris-HCl (pH 7.4), 150 mM NaCl, 1 mM EDTA, 1% (v/v) Triton X-100, 1 mM PMSF, and one cOmplete protease inhibitor tablet/50 ml) at 4°C for 30 min, followed by clarification by centrifugation at 17,000*g* for 10 min. Lysates were precleared on empty glutathione-Sepharose beads for 30 min before a 2-hour incubation with TBC1D23-coated beads. Beads were washed extensively in lysis buffer, and proteins eluted first in a high-salt elution buffer (25 mM tris-HCl, 1.5 M NaCl, and 1 mM EDTA) to release interacting proteins and then in SDS sample buffer to release the GST-fusion proteins. Proteins were precipitated from the high-salt elution sample by chloroform/methanol precipitation and resuspended in SDS sample buffer containing 1 mM 2-mercaptoethanol. Eluates were separated by SDS-PAGE and analyzed by MS.

### Affinity chromatography of 293T cell lysate with GST-tail fusions

The approach used was as described previously (*46*), as follows. Clarified lysates from 450 ml of 2× TY cultures containing bacteria expressing recombinant GST, GST-CPD (1321 to 1380), GST-CIMPR (2327 to 2491), and GST-TBC1D23 (559 to 684) were thawed. For each GST-tagged bait, 100 μl of glutathione-Sepharose 4B bead slurry (GE Healthcare) was used. Clarified bacterial lysates were added to the empty glutathione-Sepharose beads and incubated at 4°C for one hour on a roller. 293T cells (from four confluent T175 flasks per GST-tagged bait) were collected by scraping, washed twice with PBS, and lysed with lysis buffer [50 mM tris (pH 7.4), 150 mM NaCl, 1 mM EDTA, 5 mM 2-mercaptoethanol, 1% Triton X-100, and EDTA-free cOmplete protease inhibitor]. The lysate was clarified by centrifugation for 5 min at 17,000*g* and precleared with 100 μl of bead slurry for 1 hour at 4°C on a tube roller. Beads loaded with recombinant GST-tagged baits were washed once with ice-cold lysis buffer, once with lysis buffer supplemented with 500 mM NaCl, and once again with lysis buffer. Beads were incubated with the precleared 293T cell lysate for 2 to 4 hours on a roller at 4°C. Beads were washed twice with lysis buffer, transferred to 0.8 ml centrifuge columns (Pierce, 89869B), and washed twice more. Columns were brought to room temperature and eluted five times with 100 μl of elution buffer (1.5 M NaCl in lysis buffer) by centrifugation at 100*g* for 1 min; for the final elution, the sample was centrifuged at 17,000*g* for 1 min. Eluates were pooled together and concentrated to ~75 μl using a centrifugal filter (Amicon Ultra 0.5-ml 3000, Millipore, UFC500324), supplemented with 25 μl of NuPAGE 4× LDS sample buffer (Invitrogen, NP0007) containing 100 mM DTT. The eluate (40%) was separated by SDS-PAGE and stained with InstantBlue Coomassie stain (Abcam, ab119211). Each lane was cut into eight gel slices, transferred into a 96-well microtiter plate, and subjected to MS analysis.

### MS analysis of eluates from affinity chromatography

For samples prepared with GST fusions to parts of TBC1D23, slices were destained with 50% (v/v) of acetonitrile and 50 mM ammonium bicarbonate, reduced with 10 mM DTT, and alkylated with 55 mM iodoacetamide. After alkylation, proteins were digested with trypsin (Promega, UK) overnight at 37°C at an enzyme to protein ratio of 1:20. The resulting peptides were separated by nanoscale capillary liquid chromatography (LC)–MS/MS using an Ultimate U3000 high-performance LC (HPLC) (Thermo Fisher Scientific, Dionex, San Jose, USA) to deliver a flow of ~300 nl/min. A C18 Acclaim PepMap100 5 μm, 100-μm × 20-mm nanoViper (Thermo Fisher Scientific, Dionex, San Jose, USA), trapped the peptides before separation on a 25-cm PicoCHIP nanospray column packed with Reprosil-PUR C18 AQ (New Objective Inc., Littleton, USA). Peptides were eluted with a 60-min gradient of acetonitrile [2 to 80% (v/v)]. The analytical column outlet was directly interfaced via a nanoflow electrospray ionization source, with a hybrid dual pressure linear ion trap mass spectrometer (Orbitrap Velos, Thermo Fisher Scientific, San Jose, USA). Data-dependent analysis was carried out, using a resolution of 30,000 for the full MS spectrum, followed by 10 MS/MS spectra in the linear ion trap. MS spectra were collected over a mass/charge ratio (*m*/*z*) range of 300 to 2000. MS/MS scans were collected using a threshold energy of 35 for collision induced dissociation. LC-MS/MS data were then searched against a protein database (UniProtKB) using the Mascot search engine programme (Matrix Science) (*47*). Database search parameters were set with a precursor tolerance of 5 parts per million (ppm) and a fragment ion mass tolerance of 0.8 Da. Two missed enzyme cleavages were allowed, and variable modifications for oxidized methionine, carbamidomethyl cysteine, pyroglutamic acid, phosphorylated serine, threonine, and tyrosine were included. MS/MS data were validated using the Scaffold programme (Proteome Software Inc.) (*48*).

For samples prepared with GST fusions to tails of cargo proteins, gel slices (1 to 2 mm) were placed in 96-well microtiter plates and destained with 50% (v/v) of acetonitrile and 50 mM ammonium bicarbonate, reduced with 10 mM DTT, and alkylated with 55 mM iodoacetamide. After alkylation, proteins were digested with trypsin (6 ng/μl; Promega, UK) overnight at 37°C. The resulting peptides were extracted in 2% (v/v) of formic acid and 2% (v/v) acetonitrile. The digests were separated by nanoscale capillary LC-MS/MS using an Ultimate U3000 HPLC (Thermo Fisher Scientific, Dionex, San Jose, USA) to deliver a flow of ~300 nl/min. A C18 Acclaim PepMap100 5 μm, 100-μm × 20-mm nanoViper (Thermo Fisher Scientific, Dionex, San Jose, USA), trapped the peptides before separation on a C18 BEH130 1.7 μm, 75-μm × 250-mm analytical ultrahigh-performance LC column (Waters, UK). Peptides were eluted with a 60-min gradient of acetonitrile (2 to 80%). The analytical column outlet was directly interfaced via a nanoflow electrospray ionization source, with a quadrupole Orbitrap mass spectrometer (Q-Exactive HFX, Thermo Fisher Scientific). MS data were acquired in data-dependent mode using a top 10 method, where ions with a precursor charge state of 1+ were excluded. High-resolution full scans (*R* = 60,000; *m*/*z*, 300 to 1800) were recorded in the Orbitrap, followed by higher energy collision dissociation (26% normalized collision energy) of the 10 most intense MS peaks. The fragment ion spectra were acquired at a resolution of 15,000, and dynamic exclusion window of 20 s was applied. Raw data files from LC-MS/MS data were processed using Proteome Discoverer v2.1 (Thermo Fisher Scientific) and then searched against a human protein database (UniProtKB, reviewed) using the Mascot search engine programme. Database search parameters were set with a precursor tolerance of 10 ppm and a fragment ion mass tolerance of 0.2 Da. One missed enzyme cleavage was allowed, and variable modifications for oxidized methionine, carbamidomethyl cysteine, pyroglutamic acid, phosphorylated serine, threonine, and tyrosine were included. MS/MS data were validated using Scaffold. For the analysis of mass spectral intensities, all raw files were processed with MaxQuant v1.5.5.1 using standard settings and searched against UniProt with the Andromeda search engine integrated into the MaxQuant software suite (*49*). Enzyme search specificity was Trypsin/P for both endoproteinases. Up to two missed cleavages for each peptide were allowed. Carbamidomethylation of cysteines was set as fixed modification with oxidized methionine and protein *N*-acetylation considered as variable modifications. The search was performed with an initial mass tolerance of 6 ppm for the precursor ion and 0.5 Da for MS/MS spectra. The false discovery rate was fixed at 1% at the peptide and protein level. Statistical analysis was carried out using the Perseus module of MaxQuant (*50*). Peptides mapped to known contaminants and reverse hits were removed, and only protein groups identified with at least two peptides, one of which was unique, and two quantitation events were considered for data analysis. Each protein had to be detected in at least two of the three replicates. Missing values were imputed by values simulating noise using the Perseus’ default settings. To calculate *P* values, *t* tests were performed.

### Expression of ^15^N-labeled CPD(1321 to 1380) for NMR analysis

The GST-CPD (1321 to 1380) fusion contains a PreScission protease cleavage site downstream of the GST tag. An overnight 2× TY starter culture was inoculated into 1-liter flasks of NH_4_Cl-free M9 medium containing yeast nitrogen base (1.7 g/liter; Sigma-Aldrich, Y1251), ^15^NH_4_Cl (1 g/liter; Sigma-Aldrich, 299251), and glucose (4 g/liter). Protein expression was induced using 300 μM IPTG and incubated overnight at 16°C for 16 hours, and bacterial lysate was prepared as described above for nonisotopically labeled GST fusions. Excess of glutathione-Sepharose beads (500 μl of bead slurry per liter of 2× TY culture) was incubated with the clarified bacterial lysate for 1 hour at 4°C. Beads were then washed twice with lysis buffer and incubated with the clarified lysate for 1 hour at 4°C on a rotator. Beads were then washed twice with lysis buffer [50 mM tris (pH 7.4), 150 mM NaCl, 1 mM EDTA, and 1% Triton X-100] and twice with NMR buffer [50 mM tris (pH 7.4), 150 mM NaCl, and 5 mM 2-mercaptoethanol]. Beads were then incubated with GST-tagged PreScission protease (GE Healthcare, GE27-0843-01) at a concentration of 50 μl of enzyme per 1 ml of bead slurry and incubated from 5 hours to overnight at 4°C on a rotator. The supernatant was collected, concentrated using an Amicon 0.5-ml 3 KDa centrifugal filter (Millipore, UFC500324), and flash-frozen in liquid nitrogen. Protein concentration was measured using Bradford reagent (Bio-Rad, 5000006).

### NMR data collection and analysis for CPD

All CPD NMR datasets for were collected at 278 K using a 600-MHz Bruker Avance III spectrometer with a 5-mm TCI triple resonance cryoprobe. All samples were prepared with 5% D_2_O as a lock solvent, at pH 7.4, 50 mM tris, 150 mM NaCl, and 5 mM 2-mercaptoethanol. ^1^H-^15^N band-selective excitation short transient-transverse relaxation optimized spectroscopy (BEST-TROSY) was collected for all samples using an optimized pulse sequence (*51*). The assignment of backbone NH, N, Cα, Cβ, and C′ resonances of the 86 μM ^15^N-^13^C CPD (residues 1321 to 1380) sample was completed using standard three-dimensional datasets acquired as pairs to provide own and preceding carbon connectivities and between 20 and 40% nonuniform sampling to aid faster data acquisition. Both the HNCO and HN(CA)CO experimental pair and the CBCA(CO)NH and HNCACB pair were collected with 1024, 64, and 96 complex points in the proton, nitrogen, and carbon dimensions, respectively. Nitrogen connectivities were established using (H)N(COCA)NNH and (H)N(CA)NNH experiments with 2048, 64, and 96 complex points in the proton, direct, and indirect nitrogen dimensions, respectively. All data were processed using Topspin versions 3.2 or 4 (Bruker) or, if required, NMRPipe (*52*), with compressed sensing for data reconstruction (*53*), and analyzed using NMRFAM-Sparky. The backbone assignment was completed using the Mars program (*54*). Binding of TBC1D23 to the CPD construct was observed by ^1^H-^15^N BEST-TROSY. Unlabeled TBC1D23 was added to ^15^N-labeled CPD with a final concentration of both proteins at 38 μM. The ^1^H-^15^N BEST-TROSY spectrum was compared to a second spectrum recorded for a 38 μM sample of free ^15^N CPD. While some small-peak perturbations were observed, the main differences caused by TBC1D23 binding were line broadening. This was quantified by taking the peak height ratios of the bound and free spectra. To analyze the chemical shift perturbations, the following equation was used to report calculated the distance between each peak in the bound spectrum when compared to the assigned, free CPD: Δδ“total” = ((δH)^2^+ (δN/5)^2^)^0.5^, with the smallest value reported as the minimal chemical shift perturbation.

### Isothermal titration microcalorimetry

Experiments were performed using a Nano ITC machine from TA Instruments. TBC1D23 C-terminal domains were gel-filtered into buffer composed of 100 mM NaCl, 100 mM tris (pH 7.4), and 1 mM tris(2-carboxyethyl)phosphine (TCEP). Peptides were dissolved in the same batch of buffer. Both the wild-type and mutant C-terminal domains were concentrated to 100 μM (1.4 mg/ml) with peptide concentration varying between 0.8 and 5 mM (depending on the peptide). Peptides were titrated into TBC1D23 C-terminal domains with 20 injections of 2.4 μl each separated by 300-s intervals. Experiments were conducted at 20°C. A relevant syringe-peptide-into-buffer blank was subtracted from all data, and for constructs that displayed measurable binding, three independent runs that showed clear saturation of binding were used to calculate the mean *K*_D_ of the reaction, the stoichiometry (*n*), and the SEM calculated using NanoAnalyze. Data were exported to GraphPad Prism for figure generation.

### Protein expression and purification for structural analysis

Recombinant proteins were expressed in BL21 plyS *E. coli* grown in shaking 2× TY medium at 37°C. Expression was induced with 0.2 mM IPTG, and cells were grown overnight at 22°C. Cells were resuspended in buffer A [250 mM NaCl, 20 mM tris (pH 7.4), and 1 mM DTT] supplemented with 4-benzenesulfonyl fluoride hydrochloride, MnCl_2_ and deoxyribonuclease I. Cell pellets were lysed using a cell disruptor (Constant Systems) before clarification by ultracentrifugation (104,350 relative centrifugal force) for 45 min. The supernatant was batch bound to glutathione-Sepharose resin (Cytiva) in buffer A before washing with 400 ml of buffer A and overnight cleavage of the GST-tag with thrombin at room temperature. The resultant flow through was concentrated for gel filtration Superdex 200 column (GE Healthcare) into either buffer A for crystallization or into ITC buffer [100 mM NaCl, 100 mM tris (pH 7.4), and 1 mM TCEP].

### X-ray crystallography

TBC1D23 C-terminal domains was concentrated to either 11 mg/ml (full length) or 14 mg/ml (-VLDALES`) and mixed with 1.5 times molar excess syntaxin-16_209–221_ peptide. High-throughput sitting drops were used to obtain crystallization conditions, which were further optimized before being repeated in hanging drop with protein:peptide complex being mixed 1:1 ratio with crystallization mother liquor. TBC1D23 full-length C-terminal domain was crystallized in 0.02 M citric acid, 0.08 M bis-tris propane (pH 8.8), and 16% (w/v) of PEG3350. TBC1D23 (-VLDALES) C-terminal domain was crystallized in 0.8 M potassium phosphate dibasic, 0.1 M Hepes/NaOH (pH 7.5), 0.8 M sodium phosphate monobasic, and 1% of 1,2-butandiol. Crystals were cryoprotected by soaking in mother liquor supplemented with 35% glycerol and syntaxin-16_209–221_ peptide (1 mg/ml) and flash-cooled in liquid nitrogen.

Diffraction data were collected at the Diamond Light Source on the IO4 beamline at 100 K and processed with Autoproc. Structures were solved by molecular replacement with the PHASER-MR program using as a search model a single copy of the TBC1D23 C-terminal domain (6JM5) with zinc and water molecules removed. REFMAC5 was used for iterative rounds of refinement interspersed by manual rebuilding of the model using Coot. Crystallographic programs were run using the CCP4i2 package, and figures were rendered using UCSF ChimeraX (*55*). Data collection and refinement statistics are summarized in table S1. Electron density of the full-length TBC1D23 domain structure in the presence of syntaxin-16 peptide clearly showed C-terminal tails bound in two molecules and syntaxin-16 bound in a third with the fourth being somewhat ambiguous (fig. S4). Deletion of the C-terminal tail (VLDALES) and inclusion of syntaxin-16 peptide resulted in a structure whose electron density, when solved by molecular replacement using 6JM5 minus the VLDALES as the search model, unambiguously showed the syntaxin-16 peptide bound in all molecules. Coordinates and reflection data are deposited in the Protein Data Bank (PDB) under accession code 8QQF.
