# Supplementary material for: Cargo selective vesicle tethering: The structural basis for binding of specific cargo proteins by the Golgi tether component TBC1D23
Source: Sci Adv. 2024 Mar 29;10(13):eadl0608. doi: 10.1126/sciadv.adl0608 (PMC11093223; doi:10.1126/sciadv.adl0608)
Supplement: Supplementary file 1 — Figs. S1 to S5 Tables S1 and S2 Legends for data S1 to S3 [file sciadv.adl0608_sm.pdf]

Science Advances
AAAS

Supplementary Materials for

Cargo selective vesicle tethering: The structural basis for binding of specific cargo proteins by the Golgi tether component TBC1D23

Jérôme Cattin-Ortolá *et al.*

Corresponding author: David J. Owen, djo@30cam.ac.uk; Sean Munro, sean@mrc-lmb.cam.ac.uk

Sci. Adv. **10**, eadl0608 (2024)
DOI: 10.1126/sciadv.adl0608

The PDF file includes:

Figs. S1 to S5
Tables S1 and S2
Legends for data S1 to S3

Other Supplementary Material for this manuscript includes the following:

Data S1 to S3

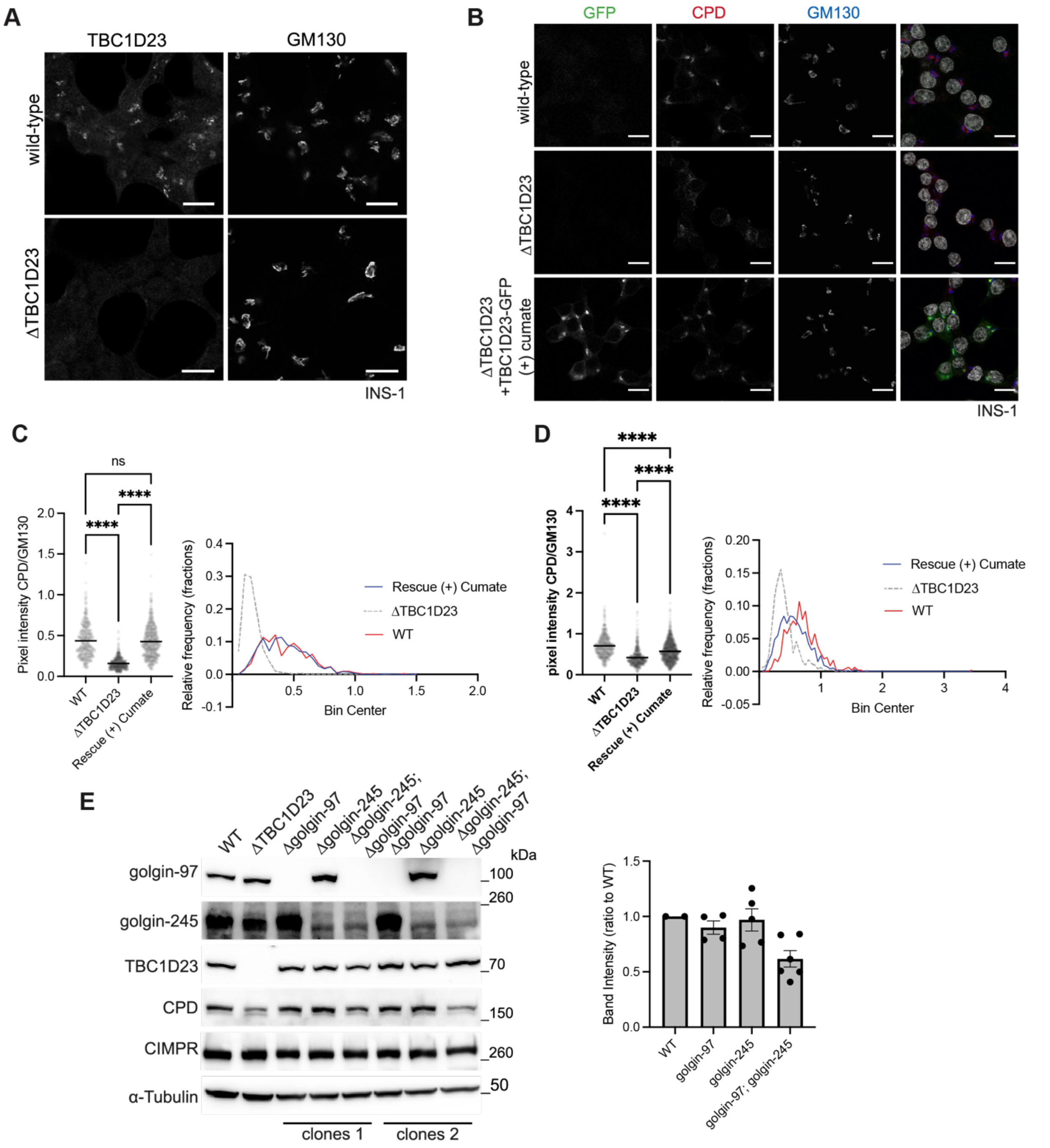

Fig. S1. Normal CPD trafficking requires TBC1D23.

(**A**) Confocal micrographs of wild-type and Δ*TBC1D23* INS-1 cells, labelled for endogenous TBC1D23 and GM130 (Golgi). Scale bar: 10 µm. Representative of three repeats. (**B**) Confocal micrographs of the indicated INS-1 cell lines, labelled for GFP-booster and endogenous CPD and GM130 (Golgi). Scale bar: 10 µm. Representative of three repeats. (**C**) Scatter plot showing the ratio of the Golgi-area fluorescence intensity of CPD over GM130 for cells as in (B). Golgi-area were detected automatically using an ImageJ macro. The black bar is mean, and for wild

type (WT), n = 422 cells; for Δ*TBC1D23*, n=521; for rescue, n=573. Statistics: ordinary one-way ANOVA followed by a Sidak's multiple comparison tests with a single pooled variance. ****: P<0.0001, ns: not significant. Also shown are the ratios plotted as frequency distributions with a bin width of 0.05. Source data in data S2. (**D**) An independent biological repeat of the experiment shown in (C). For wild type (WT), n = 468; for Δ*TBC1D23*, n = 501; for rescue, n = 946. Source data in data S2. (**E**) Immunoblot analysis of detergent lysates from HEK-293 (WT), Δ*TBC1D23*, two clones of Δ*golgin-97*, three clones of Δ*golgin-245*, and four clones of Δ*golgin-97*;Δ*golgin-245* (one or two replicates for each). On the right is a representative immunoblot, and all data summarised in the plot on the left. Total protein concentration was measured so equal amounts of protein were loaded in each lane with band intensity quantification of the CPD blot normalised to wild type for each blot. Multiple clones were tested to demonstrate reproducibility rather than pool the observations. Source data in data S2.

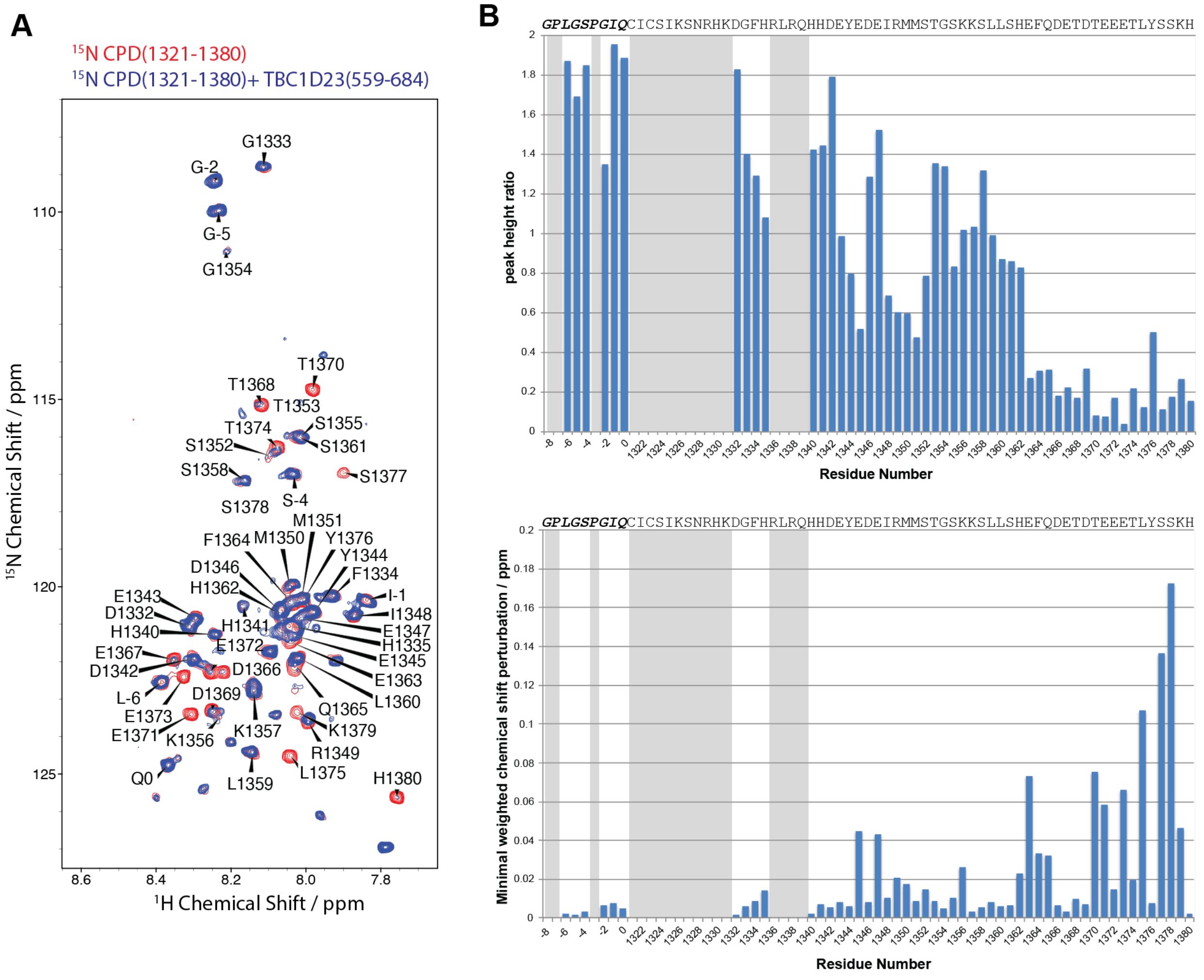

Fig. S2. NMR analysis of CPD cytoplasmic tail in presence of TBC1D23 C-terminal domain.
(**A**) The assigned ^{1}H-^{15}N BEST-TROSY of ^{15}N labelled CPD (residues 1321-1380) is shown in red overlaid with the BEST-TROSY of ^{15}N CPD with unlabelled TBC1D23 (residues 559-684) at a 1:1 ratio shown in blue. Both spectra were collected at 278 K and 600 MHz. (**B**) Peak height intensity differences of the ^{15}N CPD peaks in the presence and absence of unlabelled TBC1D23 are reported as a ratio (top panel). The minimal chemical shift perturbations seen for ^{15}N CPD in the presence and absence of unlabelled TBC1D23 (bottom panel). Grey areas indicate missing assignments.

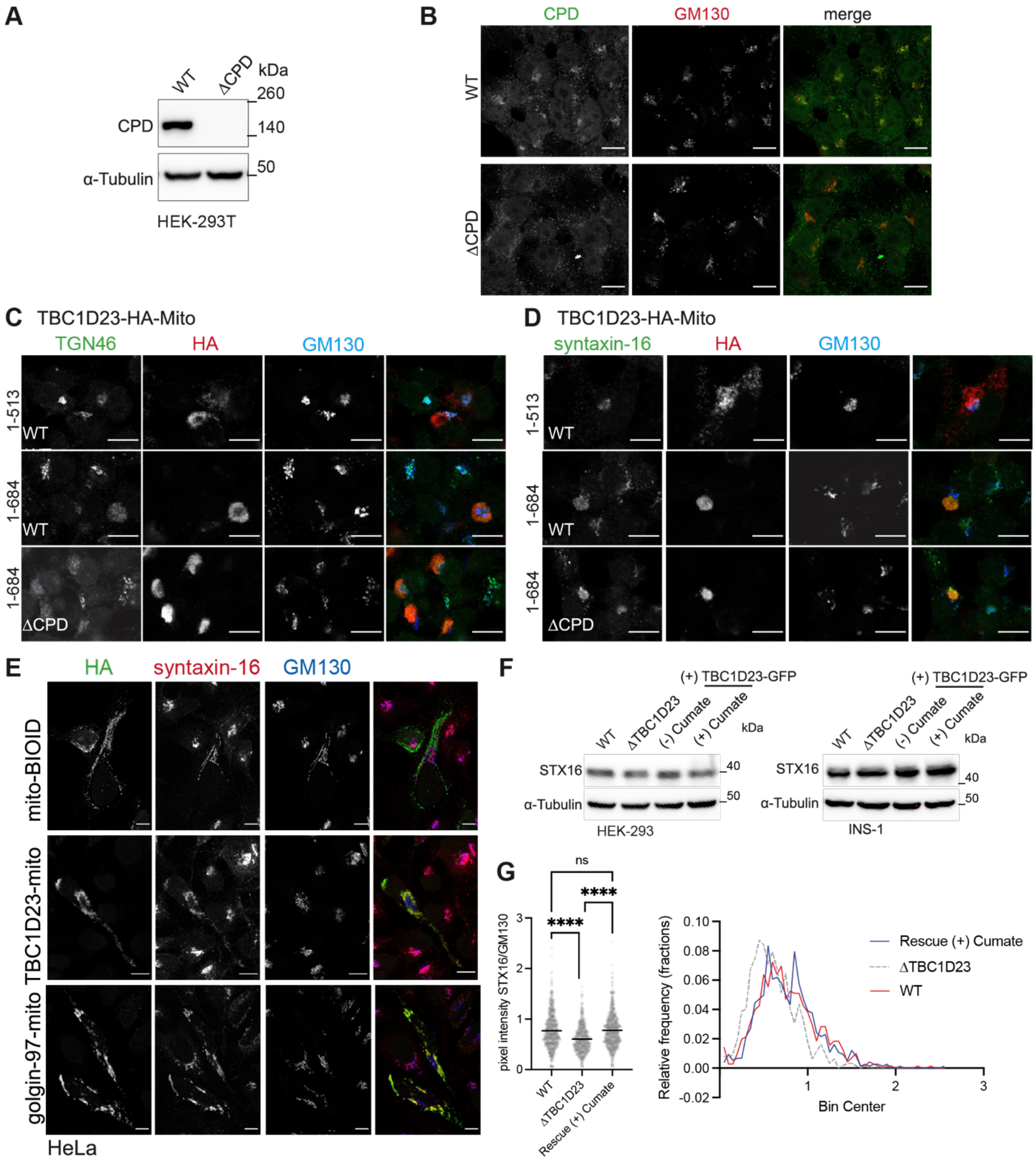

Fig. S3. CPD is not required for TBC1D23 to capture vesicles.
(**A**) Immunoblots of detergent lysates from 293T wild type (WT) and Δ*CPD*. Cells were lysed with Triton-X-100. Total protein concentration was measured so equal amount of protein was loaded in each lane. (bottom) band intensity quantification of the CPD blot normalised to WT for each blot. (**B**) Confocal micrographs of wild type (WT) and Δ*CPD* 293T cells. Cells were fixed, permeabilised and stained using endogenous CPD and GM130 (Golgi marker). Scale bar: 10 µm.

Representative of two repeats. (**C**) Confocal micrographs of HeLa cells TBC1D23-mito chimaeras and labelled for the HA tag in the chimaera, endogenous TGN46 and GM130. Scale bar: 10 µm. Representative of two repeats.(**D**) Confocal micrographs of HeLa cells TBC1D23-mito chimaeras and labelled for the HA tag in the chimaera, endogenous syntaxin-16 and GM130. Scale bar: 10 µm. Representative of two repeats. (**E**) Confocal micrographs of HeLa cells expressing golgin-97-mito or TBC1D23-mito chimaeras and labelled for the HA tag in the chimaera and endogenous syntaxin-16 and GM130. Mito-BioID was used as a negative control. Scale bar: 10 µm. (**F**) Immunoblots of detergent lysates from HEK-293 or INS-1 cells as indicated. WT, Δ*TBC1D23*, or rescue Δ*TBC1D23* cells stably expressing TBC1D23-GFP under a cumate promoter with the cells incubated in the absence (-) and in presence (+) of cumate for 24-36 hours. These are the samples as in Fig. 2A, but here are blotted for syntaxin-16. (**G**) As Fig. 3I: independent biological repeat for syntaxin-16 Golgi localisation. For WT, n = 705; for Δ*TBC1D23*, n = 619; for the stable rescues, n = 743. Source data for fig. S3G are in data S2.

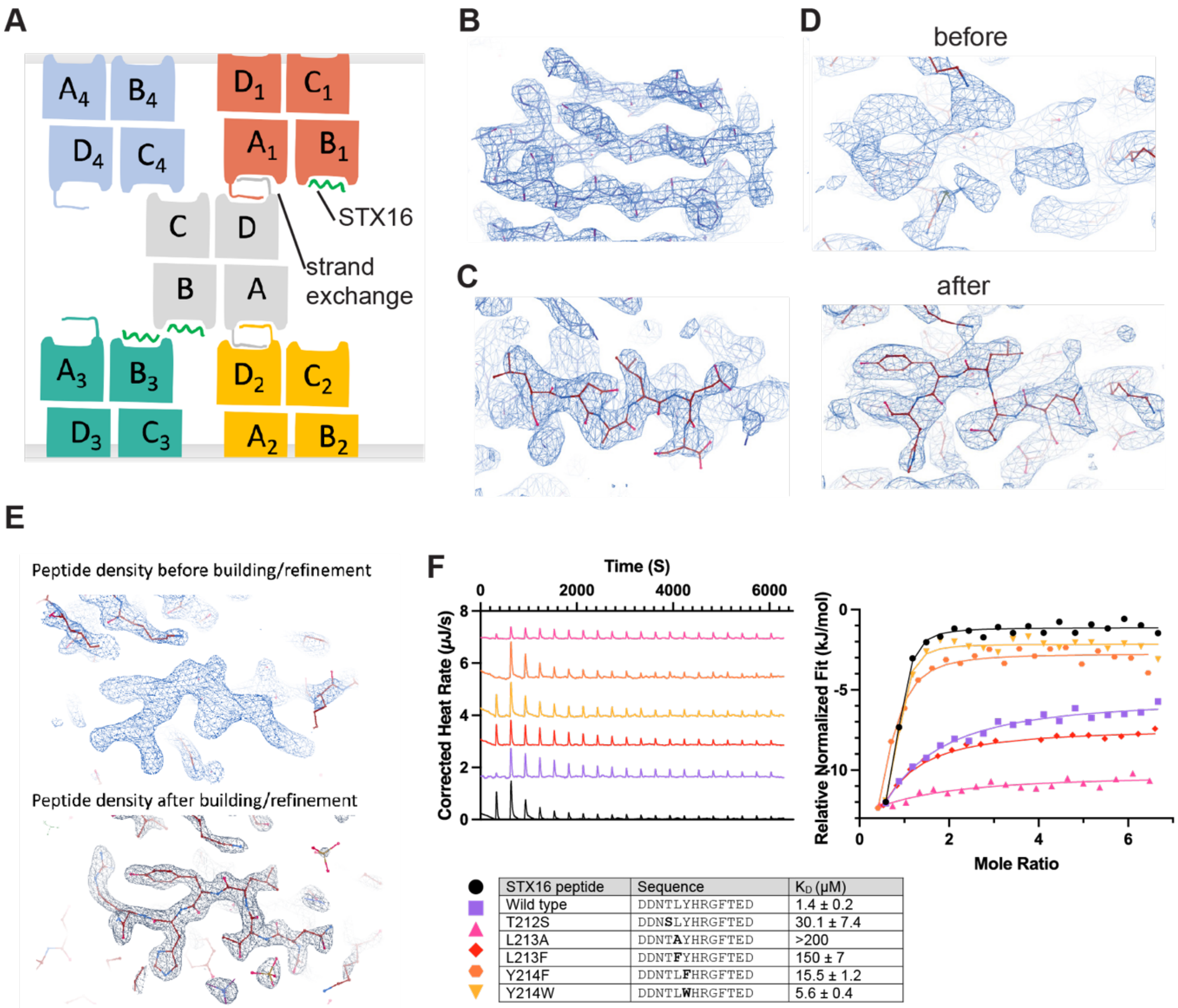

STX16 peptide	Sequence	K_D (μM)
Wild type	DDNTLYHRGFTED	1.4 ± 0.2
T212S	DDN**S**LYHRGFTED	30.1 ± 7.4
L213A	DDNT**A**YHRGFTED	>200
L213F	DDNT**F**YHRGFTED	150 ± 7
Y214F	DDNTL**F**HRGFTED	15.5 ± 1.2
Y214W	DDNTL**W**HRGFTED	5.6 ± 0.4

Fig. S4. Structural and biochemical characterisation of the interaction between the TBC1D23 C-terminal domain and the syntaxin-16 peptide.
(**A**) Cartoon of the crystal packing of the four copies of the TBC1D23 C-terminal domain showing the C-terminal strand exchange that occurs for copies A and D, with the syntaxin-16 peptide bound to copy B, and the occupancy of copy C unclear. (**B**) Representative electron density of full-length TBC1D23 C-terminal domain contoured at 2σ. (**C**) Electron density of TBC1D23 full length tail (..MKVLDALES) blocking the syntaxin-16 binding site, as seen for copies A and D in (A). (**D**) Electron density of the syntaxin-16 peptide bound to the TBC1D23 full length C-terminal domain (copy B in (A)), shown before and after building and refinement. (**E**) Density map showing the initial experimental density (blue) obtained with MR for the C-terminal lacking the last eight residues domain with syntaxin-16 peptide bound, and the final 2Fo-Fc electron density (black) with syntaxin-16 peptide built in. (**F**) ITC of relative binding of mutated forms of the syntaxin-16 peptide to the TBC1D23 C-terminal domain. Affinity calculated from three repeats – see data S2.

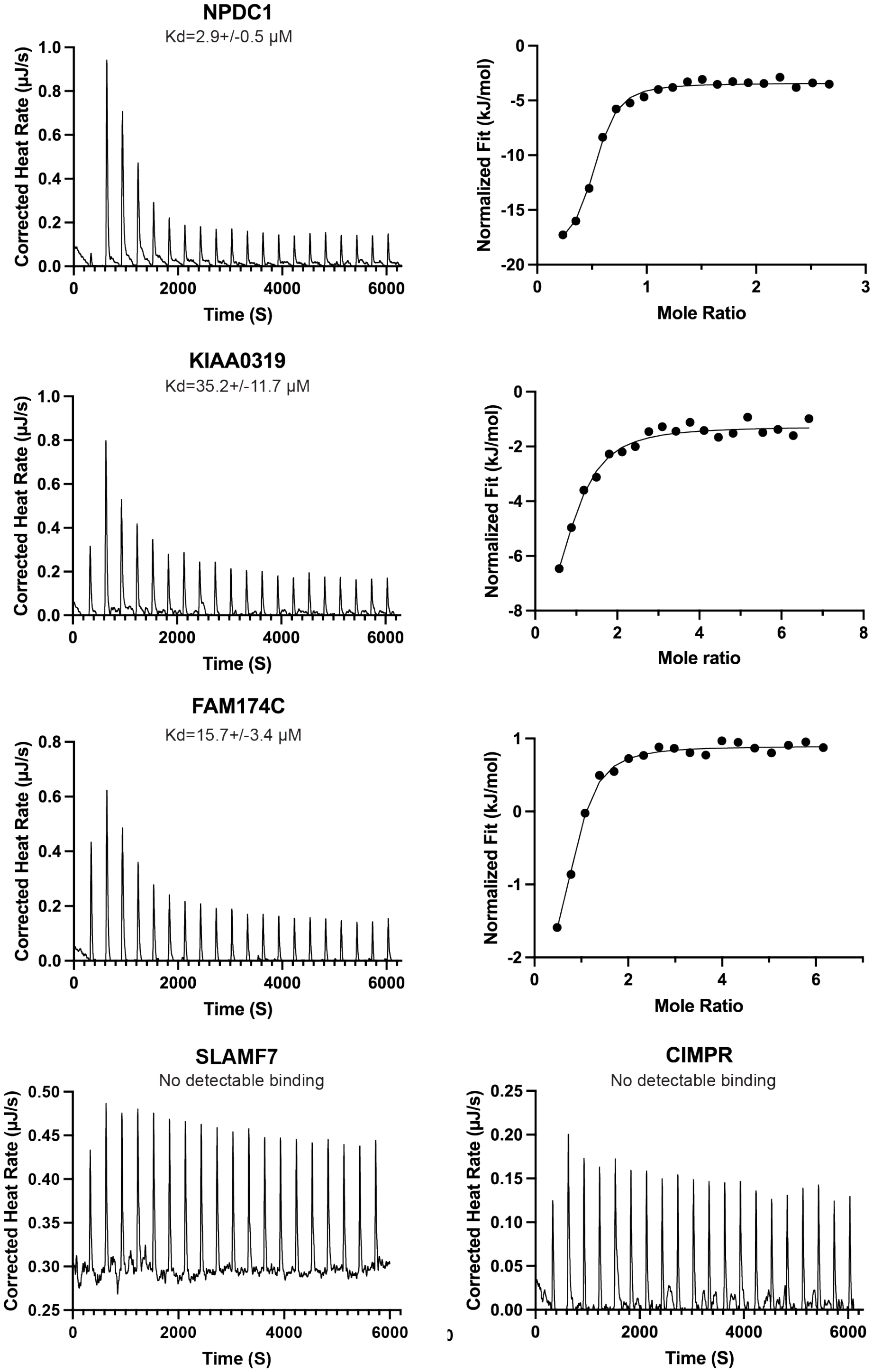

Fig. S5. Binding of peptides containing the acidic TLY motif from several proteins that recycle between endosomes and Golgi.

ITC of the binding to the TBC1D23 C-terminal domain of peptides corresponding to TLY-motif containing regions from the cytoplasmic tails of the indicated proteins. Affinity calculated from three repeats – see data S2.

	Mouse TBC1D23 (559-684)	Mouse TBC1D23 (559-677) with human STX16 (206-221)
PDB accession		8QQF
Space group	P 62 2 2	P 65 2 2
Resolution range	194.38-3.17	116.19-2.19
InnerShell	112.394-11.533	116.19-8.280
OuterShell	3.814-3.172	2.479-2.187
Rmerge (all I+ & I-)	0.939 (2.988)	0.133 (2.607)
Rmerge (within I+ & I-)	0.989 (2.998)	0.141 (2.710)
Rmeas (all I+ & I-)	0.951 (3.028)	0.136 (2.651)
Rmeas (within I+ & I-)	1.013 (3.073)	0.145 (2.791)
Rpim (all I+ & I-)	0.151 (0.485)	0.024 (0.472)
Rpim (within I+ & I-)	0.220 (0.667)	0.034 (0.668)
Number of observations	352792 (17313)	486437 (23072)
Number of unique observations	9118 (457)	14895 (745)
<(I)/sd(I)>	7.5 (1.9)	23.0 (1.9)
CC(1/2)	0.972 (0.627)	0.999 (0.707)
Completeness % (spherical)	90.8 (47.7)	43.2 (7.0)
Completeness % (ellipsoidal)	90.8 (47.7)	94.8 (82.9)
Multiplicity	38.7 (37.9)	32.7 (31.0)
RMS bond lengths (Å)	0.0063	0.0077
RMS bond angles (°)	1.644	1.605
Reflections (All/Free)	8038 / 356	14895/752
Rfactor/Rfree	0.209 / 0.274	0.186 / 0.259
Ramachandran (%) (favoured/outliers)	97.11/2.89	99.24/0.76

Table S1. Crystallographic data collection and refinement statistics.

UniProt ID	Gene	motif	motif_pos	terminus	tmds	tmd_topo _N;C	adj_tmd_pos
O00264	PGRMC1	KEGEEPTVY	172-180	C-term	1	oi	25-43
Q9BVV8	FAM174C	LDSDEETVF	118-126	C-term	1	oi	73-93
Q3ZCQ3	FAM174B	DEDEDSTVF	146-154	C-term	1	oi	91-111
Q8TBP5	FAM174A	DEDDDNTLF	175-183	C-term	1	oi	124-144
Q9NQX5	NPDC1	NEDGDFTVY	280-288	C-term	1	oi	182-202
Q01344	IL5RA	VETLEDSVF	412-420	C-term	1	oi	343-362
Q5VV43	KIAA0319	FDSDQDTIF	1033-1041	C-term	1	oi	956-976
O75051	PLXNA2	VDDLFETLF	1692-1700	C-term	1	oi	1238-1258
O75976	CPD	TDTEEETLY	1368-1376	C-term	1	oi	1300-1320
O14662	STX16	DDGDDNTLY	206-214	N-term	1	io	302-322
Q68D42	TMEM215	CPEPEDSIF	189-197	C-term	2	io;oi	40-60
Q96ET8	TVP23C	DDTEDVSLF	8-16	N-term	2	io;oi	52-72
Q9NYZ1	TVP23B	DDTEDVSLF	8-16	N-term	4	io;oi	34-53
Q8NFJ5	GPRC5A	FEETGDTLY	309-317	C-term	7	oi;oi	248-268
Q96FM1	PGAP3	SFLEDDSLY	300-308	C-term	7	oi;oi	280-299
Q86SP6	GPR149	YDDDENSIF	418-426	C-term	7	oi;oi	343-363
Q9NUN5	LMBRD1	ISDDEPSVY	530-538	C-term	9	oi;oi	487-507
Q96RN1	SLC26A8	KEEEIFSLF	586-594	C-term	12	io;oi	498-518
Q96QE2	SLC2A13	KLEEIESLF	601-609	C-term	12	io;oi	574-594
BioPlex 3.0 hits with TBC1D23 contain four membrane proteins:							
NPDC1							
FAM174A							
NIPAL1							
HS2ST1							

Table S2. Acidic TLY motif containing membrane proteins.

Human membrane proteins with the motif [DE]$_{>=3/5}$[TS][LIV][YFW] in a cytoplasmic domain. Also shown are the four membrane proteins reported to interact with TBC1D23 in the BioPlex protein interaction screen.

Data S1. (separate file)
The mass spectrometry data plotted in Figs. 1, B and C; and Fig. 3D.

Data S2. (separate file)
Source data for plots shown in Fig. 2, B, E and F; Fig. 3I; fig. S1, C to E; and fig. S3G; and for ITC measurements shown in Fig. 4A, Fig. 5C, Fig. 6B, fig. S4F, and fig. S5.

Data S3. (separate file)
Antibodies, plasmids and cell lines used in this study.
